# Drug repurposing: a dual-mechanism antibiotic combats MRSA and its high resistant phenotypes

**DOI:** 10.3389/fcimb.2026.1851763

**Published:** 2026-05-28

**Authors:** Pengfei She, Dan Xiao, Guanqing Huang, Shaowei Guo, Yiqing Liu, Mengna Li, Lihua Lu, Yelan Hong, Yong Wu

**Affiliations:** 1 Department of Laboratory Medicine, The Third Xiangya Hospital of Central South University, Changsha, China; 2 Department of Laboratory Medicine, The First Hospital of Changsha (The Affiliated Changsha Hospital of Xiangya School of Medicine, Central South University), Changsha, China; 3 Graduate Collaborative Training Base of The First Hospital of Changsha, of Hengyang Medical College, University of South China, Hengyang, China; 4 Department of Laboratory Medicine, The Xiangya Hospital of Central South University, Changsha, China

**Keywords:** antimicrobial agents, biofilms, drug repurposing, *in vivo*, methicillin-resistant *Staphylococcus aureus*, peptidoglycan, proton motive force

## Abstract

**Purpose:**

Methicillin-resistant *Staphylococcus aureus* (MRSA) is frequently observed in biofilm form, exhibiting significant tolerance, particularly in the context of deep tissue infections such as osteomyelitis. This characteristic renders clinical treatment extremely challenging. Therefore, the development of novel antibacterial agents against MRSA and its biofilms is identified as an urgent priority.

**Methods:**

By drug repurposing, this study determines the antibacterial activity of the anti-amoebic small molecule tiliquinol against MRSA and its highly drug-resistant biofilms and evaluates its potential to induce MRSA resistance. The antibacterial mechanism of tiliquinol is examined utilizing transcriptomics, fluorescent probes, and quantitative reverse-transcription polymerase chain reaction. In addition, we evaluate *in vivo* antibacterial efficacy and safety profiles by established multiple murine infection models, including skin and soft tissue infection, sepsis, as well as the periprosthetic joint infection.

**Results:**

The research find that tiliquinol exhibits notable antibacterial activity against MRSA and its highly resistant biofilms avoiding resistance occurrence. Mechanism study reveals that the proton motive force and peptidoglycan are potential therapeutic targets by tiliquinol. Further, in various mouse infection models, favorable *in vivo* antibacterial efficacy with satisfied safety profiles of tiliquinol are confirmed.

**Conclusion:**

Tiliquinol is recognized as a novel therapeutic strategy against refractory MRSA-related infections, particularly those associated with osteomyelitis and implant-related complications.

## 1 Introduction

In recent years, *Staphylococcus aureus* has been commonly associated with increasing bacterial resistance to antibiotics worldwide. And it is currently included in the pathogens of ESKAPE group (*Enterococcus faecium*, *S. aureus*, *Klebsiella pneumoniae*, *Acinetobacter baumannii*, *Pseudomonas aeruginosa*, and *Enterobacter* species), which includes the most important bacteria involved in infections and characterized by multidrug resistance (Miller and Arias, 2024). *S. aureus* strains develop resistance mechanisms against nearly all available antibiotics, specifically *β*-lactams, glycopeptides, and oxazolidinones (Mlynarczyk-Bonikowska et al., 2022). Among which, the most prevalent resistance mechanism is mediated by the production of penicillin-binding protein PBP2a, encoded by *mecA*, owning to the resistance to *β*-lactam antibiotics (Hryniewicz and Garbacz, 2017). Numerous commonly used antibiotics, including methicillin, penicillin, oxacillin, cloxacillin, cefazolin, and cefoxitin, are ineffective against Methicillin-resistant *S. aureus* (MRSA) strains. Its drug resistance mainly stems from the *mecA* gene it carries (Mudassar et al., 2025). MRSA has become a common pathogen responsible for various infections. These range from chronic recurrent skin and soft tissue infections to deeper organization such as bone and joint infections and endocarditis, resulting in high incidence rates and mortality (Kwiecinski and Horswill, 2020; Linz et al., 2023). Although, vancomycin (VAN) remains a last-resort treatment for MRSA infections, intermediate or resistant strains have emerged globally (Hiramatsu et al., 2014).

*S. aureus*, including MRSA, is the main pathogen of biofilm-related infections (Lebeaux et al., 2013). *S. aureus* biofilm accumulation can be occurred through the action of the *icaADBC*-encoded polysaccharide intercellular adhesin or by surface proteins activation (Peng et al., 2022). Accessory gene regulatory (*agr*) operon is the main gene encoding the biofilm formation and dispersal (Idrees et al., 2021). Bacterial microcolonies and extracellular polymeric substances (EPS) are critical components of the biofilm. The polysaccharide intercellular adhesin is the major component of EPS in *S. aureus* biofilm, which is a cationic polysaccharide composed of partially deacetylated N-acetylglucosamine monomers (NAG) (Cheung et al., 2021; Kaushik et al., 2024). EPS confers antibacterial resistance to bacteria through multiple mechanisms. For example, EPS physically isolates individual bacteria from antibacterial agents, it can also indirectly facilitate the development of resistance by inhibiting bacterial metabolic activities (Kaushik et al., 2024). Skin and soft tissue infections, infective endocarditis, pneumonia, osteomyelitis, and joint infections are all closely associated with the formation of biofilms (Liu et al., 2011). Biofilms pose considerable challenges to implant-related infections, because the tough surfaces of medical apparatus often exhibit a large specific area with enhanced physical and chemical properties facilitating colony colonization and subsequent biofilm development (Kaushik et al., 2024). Consequently, the pursuit of novel antibacterial agents holds significant importance for the treatment of these infections.

As a crucial component of cell structure, the cell membrane represents a potential target for novel antibacterial development. The proton motive force (PMF) of bacteria sustains the electrochemical proton gradient across the cell membrane and maintain the stability of the cell membrane. The electric potential (ΔΨ) and transmembrane proton gradient (ΔpH) are two primary components of PMF. PMF inhibitors that can dissipate either ΔΨ or ΔpH exhibit effectively antimicrobial activity (Mohiuddin et al., 2022). Among these compounds, Halicin, an antidiabetic drug, emerges as a broad-spectrum antibacterial molecule capable of selectively dissipating ΔpH (Stokes et al., 2020). The small molecule compound JD1 exhibits bactericidal effects against MRSA and its biofilms by disrupting PMF (Dombach et al., 2021).

Repurposing drugs to combat bacterial infections offers significant advantages. In comparison to the *de novo* drug development, this approach can effectively shorten the research period and reduce costs. Recent studies highlighted the antibacterial activity of non-antibiotic agents. For example, tafenoquine, a drug for treating malaria, had a significant inhibitory effect on the planktonic bacteria, biofilms and persister cells of MRSA. Lusutrombopag, an oral thrombopoietin receptor agonist, had antibacterial activity against *Enterococcus*, with a low tendency to induce drug resistance and toxicity (She et al., 2023, 2024). Tiliquinol is a 5-methyl, 8-hydroxyquinoline compound, and the tiliquinol-tilbroquinol combination is used for the treatment of amoebic diseases (Desoubeaux et al., 2014). Additionally, previous investigations have also documented the biological activity of tiliquinol-tilbroquinol against *Vibrio cholerae* (Bougoudogo et al., 1992). However, to the best of our knowledge, the antibacterial effect of tiliquinol against *S. aureus* remains unexplored.

The present study discovered that tiliquinol exhibited antibacterial activities against MRSA and its biofilms without evidence of drug resistance occurrence. Meanwhile, the mechanistic study indicated PMF and peptidoglycan were potential targets of tiliquinol. The *in vivo* antimicrobial efficacy and toxicity profile of tiliquinol were further assessed by varied infectious models. Collectively, our research suggests that tiliquinol possessed significant potential in the treatment of MRSA and its biofilm-associated refractory infections.

## 2 Materials and methods

### 2.1 Bacterial strains and cell lines, culture conditions

Type strains of *S. aureus* (ATCC 43300, USA300, ATCC 29213, and ATCC 25923), *A. baumannii* ATCC 19606, *E. faecalis* ATCC 51299, ATCC 29212, *Escherichia coli* ATCC 25922, and *E. faecium* ATCC 19434 were purchased from the America Type Cell Center (ATCC). *S. aureus* MW2, RN4220 and RJ-2 were kindly given by Min Li (Shanghai Jiaotong University, Shanghai, P.R. China). *S. epidermidis* RP62A and ATCC 12228 were provided by Di Qu (Shanghai Medical College, Fudan University, Shanghai, P.R. China). *K. pneumoniae* ATCC 700603 was provided by Juncai Luo (Tiandiren Biotech, Changsha, P.R. China). *P. aeruginosa* PAO1 was provide by Minqiang Qiao (College of Life Sciences of Nankai University, Tianjin, P.R. China). *E. faecalis* clinical isolates of SRYEFM1, SRYEFM2, SRYEFM5, SRYEFM6, SRYEFM9, SRYEFM13 and SRYEFM15 were provided by Liangyi Xie (Hunan Provincial People’s Hospital, Changsha, P.R. China). *E. faecium* U101 and staphylococci clinical strains were isolated from patient urine and blood, respectively, at the Third Xiangya Hospital of Central South University between 2014 and 2020. All clinical strains dual identified by the VETIK 2 Compact (bioMerieux, France) as well as the matrix-assisted laser desorption ionization-time of flight mass spectrometry (Bruker, Germany). Gram-positive cocci of Enterococcus and Staphylococci strains were grown in brain-heart infusion (BHI) and trypsin soybean broth (TSB), respectively, while Gram-negative bacteria were cultured in Luria-Bertani (LB) broth. All culture media was autoclaved at 121 °C for 15min before use. Tiliquinol and other chemicals used in the study were mainly purchased from the MedChem Express (NJ, USA). All reagents and compounds were dissolved as a stock solution in dimethyl sulfoxide (DMSO) or deionized water. All bacteria were propagated at 37 °C 200 rpm. Cell lines of HEK293T, LO2, HepG2, HaCaT, HSF, A549, and RAW264.7 were cultured in RPMI medium or Dulbecco’s modified Eagle’s medium supplemented with10% fetal bovine serum.

### 2.2 Antimicrobial susceptibility test

According to the Clinical and Laboratory Standards Institute (CLSI) guidelines (Schuetz et al., 2025), multiple proportion dilutions of compounds were conducted in Mueller-Hinton (MH) broth and mixed with equal volume of isometric log-phased bacterial suspension containing to a final concentration of approximately 1.5 × 10^6^ CFU/mL. After incubation at 37 °C for 16–20 h, the concentration at which the growth turbidity of bacteria could not be observed by naked eye was defined as the minimum inhibitory concentration (MIC). And The minimum bactericidal concentration (MBC) was determined as the concentration that killed 99.9% bacterial colonies on agar plates.

### 2.3 Kirby-Bauer test

*S. aureus* ATCC 43300 was grown overnight in TSB, and adjusted to a McFarland (McF) turbidity of 0.5. Then, 100 µL of the suspension was evenly spread onto MH agar plates with a sterile cotton swab. Blank disks loaded with various amounts of compounds were placed on the agar, DMSO was used as a negative control. After incubation at 37 °C for 18h, the inhibition zones were recorded by a caliper (Hu et al., 2025).

### 2.4 Bacterial live/dead cells detection by SYTO9/propidium iodide fluorescent probes

Log-phased *S. aureus* was adjusted to OD630nm of 0.2 in 1× PBS in the presence of indicated concentrations of compounds. After incubation at 37 °C for 2h, 1 mL of the bacterial suspension was centrifuged at 3500×g for 10min. Then, the bacteria was incubated with a mixture of 10 μM SYTO9 and PI at a ratio of 1:1 (vol/vol) in the dark for 15min. After washing with PBS, the bacterial viability was observed by using a confocal laser microscope (CLSM, LSM800, ZESS, Germany) (Robertson et al., 2021).

### 2.5 Bactericidal kinetics

Log-phased *S. aureus* was adjusted to approximately 1×10^6^ CFU/mL in TSB medium. Then, tiliquinol stock solution was added to the bacterial suspension to the final concentrations of 0.25×MIC, 0.5×MIC, 1×MIC, 2×MIC and 4×MIC, respectively. DMSO (0.1%, vol/vol) was added as control. An aliquot of the bacterial suspension was collected at the time points of 0, 2, 4, 8, 12 and 24h, respectively, and the number of viable bacterial cells was determined by the plate counting method (Li et al., 2022b).

### 2.6 Multi-step resistance-inducing assay

The MIC values of tiliquinol and ciprofloxacin (CIP) against *S. aureus* were determined as described above. Then, 5 µL of the bacterial suspension at the wells of 1/2×MIC was 1: 1000 diluted into 5 mL fresh MH broth as the inoculation for the next passage MIC determination. The assay was performed for a consecutive 15 days. In addition, the MICs of tiliquinol against the CIP-induced resistant strains at the last passage were also determined as described above (Kim et al., 2018).

### 2.7 One-step resistance-inducing assay

The MH agar plates were prepared with MH II cation-adjusted broth (Solarbio, Shanghai, P.R. China) and agarose (17 g/L; BioFroxx, Germany) in the presence of 1-4 × MIC of tiliquinol or CIP. Log-phased *S. aureus* was adjusted to 1 × 10^9^ CFU/mL in saline by centrifugation and resuspension. Then, 100 μL of the bacterial suspension was spread on the prepared MH plates. After incubation at 37 °C for 48h, the bacterial colonies were counted (Smith et al., 2018).

### 2.8 Postantibiotic effects

Glass tubes containing 5 mL of MH broth in the presence of 1-4×MIC of tiliquinol or vancomycin were inoculated with 5×10^6^ CFU/mL of *S. aureus*. MH broth with 0.1% DMSO was used as a control. After incubation at 37 °C and 180 rpm for 1h, the cultures were diluted 1: 1000 to remove the effects of residual antimicrobials. Viable cell counts were determined by CFU counting at the time points of 0, 2, 4, 8, 12, and 24h, respectively (Dörr, 2021).

### 2.9 Biofilm determination by crystal violet staining

The biofilm was prepared and treated as described above. The non-adherent bacterial cells were removed by washing with 1×PBS. Then, 0.15% (wt/vol) crystal violet was added to each well. After incubation at room temperature for 15min, the plates were washed three times with 1×PBS. After drying, the dye bound to the biofilms was dissolved in 95% ethanol for 20min. The biomass of biofilms was quantified by measuring the A570nm. Similarly, conventional antibiotics were used as controls (O'Toole, 2011).

### 2.10 Biofilm determination by XTT reduction assay

Overnight *S. aureus* cultures were diluted 1: 100 in TSB containing 1% glucose (TSBg) and treated with 0.25-8 μg/mL of tiliquinol in 96-well cell plates for biofilm inhibition assay. After incubation statically at 37 °C for 24h, the supernatant was carefully removed and washed with 1×PBS. Then, 100 μL of XTT (0.2 mg/mL) and PMS (0.02 mg/mL) in 1× PBS were mixed and added to each well. The plate was incubated in the dark for 3h, and the A490nm was measured. For biofilm eradication assay, the overnight cultured *S. aureus* was diluted by TSBg at the ratio of 1: 100 and incubated for 24h in 96-well plates to pre-form a biofilm. Subsequently, the supernatant was removed and the wells were washed with 1×PBS. Two hundred microliters of TSB containing 1-32 μg/mL of tiliquinol were added to each well. After incubation at 37 °C for further 24h, the supernatant was removed and the remained biofilms were stained with XTT and the A490nm was measured as described above. In addition, conventional antibiotics of VAN, daptomycin (DAP) and linezolid (LZD) were used as the controls (Xu et al., 2021).

### 2.11 Biofilm observation by confocal laser scanning microscopy

The biofilms were prepared and treated as described above in 6 well plates in the presence of a glass cover slides in each well. After the treatment, the slides were washed with 1× PBS, and stained with 10 µM SYTO9 and PI in the dark for 15min. The residual dye was removed by PBS washing and the biofilm morphology was visualized by a CLSM (LSM800, Zeiss, Jena, Germany) (Parga et al., 2023).

### 2.12 Biofilm formation on titanium discs

To evaluate the anti-biofilm effects by tiliquinol on medical implant related materials, we attempted to establish the biofilms on Ti discs. Briefly, for biofilm inhibition, overnight cultures of *S. aureus* were diluted 1: 100 with TSBg in the presence or absence of specified concentrations of compounds. Three milliliters of the bacterial suspension were inoculated into a 6-well plate containing sterile Ti discs in each well. After incubated at 37 °C for 24h, the unattached cells were removed by 1×PBS washing, and the remained biofilms were quantified by crystal violet staining and XTT reduction assay as described above. For biofilm eradication, the biofilms on Ti discs were pre-formed for 24h, followed by the treatment of serial concentrations of compounds. After further incubated at 37 °C for 24h, the biofilms were quantified by violet staining and XTT staining as well. VAN, DAP and LZD were used as the positive controls (Xu et al., 2021).

### 2.13 Scanning electron microscopy and transmission electron microscopy

The bacteria cultures in the log phase were adjusted to 3×10^8^ CFU/mL in fresh TSB containing 10×MIC of tiliquinol. Meanwhile, 0.1% DMSO was used as a control. After incubation at 37 °C for 30min. The bacterial cells were fixed with 1.5 mL of 2.5% glutaraldehyde at 4 °C for 48h. After washing twice with PBS, the samples were dehydrated through a graded ethanol series (30, 50, 70, 80, 90 and 100%). Finally, the samples were coated with gold-palladium for observation using SEM (Hitachi, Japan). The samples were dehydrated by the above methods, Then, a 1-hour incubation in propylene oxide, the samples were infiltrated with a 1:1 (vol/vol) mixture of propylene oxide and Epon for another hour. Finally, the specimens were polymerized at a temperature of 60 °C for a period of 48h. Ultrathin sections (60 nm) cut using an ultramicrotome (Reichert Ultracut-S) and stained with lead citrate. Finally, the cells were observed using TEM (Hitachi, Japan) (Belley et al., 2009; Zhang et al., 2021).

### 2.14 SYTOX green tracing

Log-phased *S. aureus* ATCC 43300 was collected by centrifuged at 4000×g for 15min, washed three times with 1×PBS (pH = 7.4) and resuspended to OD630nm = 0.5. Then, STYOX Green was added to the suspension to a final concentration of 2 μM with incubation in the dark for 30min. Fifty microliters of the labeled bacterial suspension with 50 μL of indicated concentrations of tiliquinol were incubated in a black 96-well plate. DMSO (0.1%, vol/vol) and 4 μg/mL melittin were served as the negative and positive control, respectively. The fluorescence intensities were detected by the multimode microplate reader at the excitation/emission wavelengths of 485 nm/525 nm, respectively (She et al., 2023).

### 2.15 PMF determination by DiSC3(5) probe

DiSC3(5) is a fluorescent probe which reflects the disruption of transmembrane potential component of PMF. The log-phased bacterial suspension was adjusted to OD630nm = 0.3 in 5 mM HEPES buffer containing 5 mM glucose and 100 mM KCl, then added with 2 μM of DiSC3(5). After incubation at 37 °C for 1h, the bacterial suspension was treated with 1/4, 1/2, 1 and 2×MIC of tiliquinol with 0.1% (vol/vol) DMSO as the control. The luminescence intensities were measured by a microplate reader (PerkinElmer EnVision; USA) with the excitation/emission wavelengths of 622 nm/670 nm, respectively (Farha et al., 2013).

### 2.16 PMF determination by BCECF-AM probe

The transmembrane proton gradient of PMF was determined by BCECF-AM, a pH-sensitive fluorescent probe. Briefly, the log-phased bacterial suspension was adjusted to OD630 = 0.1 in 5 mM HEPES buffer. The bacterial suspension was mixed with 10 μM BCECF-AM probe and incubated for 30min, and then treated with indicated concentrations of tiliquinol. In addition, 0.1% (vol/vol) DMSO was used as the control. The luminescence intensity was measured by using the microplate reader at an excitation/emission wavelength of 488 nm/535 nm, respectively (Liu et al., 2020).

### 2.17 PMF disruption detection by pH-adjusted MH broth

The pH gradient, ranging from 4-9, of the MH broth was adjusted by addition of concentrated HCl or NaOH. Mid log-phased *S. aureus* was diluted to approximate 1×10^6^ CFU/mL in pH-adjusted broth with or without a series of concentrations of tiliquinol. Then, the bacterial suspension was incubated at 37 °C 180 rpm, and the turbidity was measured by OD630nm at the time points of 0, 4, 8, 12, and 24h, respectively (Li et al., 2023).

### 2.18 Drug combination assay

The combined antibacterial effects between tiliquinol and conventional antibiotics were evaluated by using the checkerboard dilution assay. Briefly, overnight cultures of *S. aureus* were diluted in MH broth to approximately 1×10^6^ CFU/mL. The compounds were two-fold serially diluted with MH broth, 50 μL of each dilution were added vertically and horizontally into individual wells of a 96-well plate in the presence of prepared bacterial suspension. The plate was then incubated at 37 °C for 16–18 h, followed by measurement of the OD630nm. The fractional inhibitory concentration index (FICI) was calculated as (MIC_A in combination_/MIC_A alone_) + (MIC_B in combination_/MIC_B alone_) (Zhao et al., 2016).

### 2.19 Intracellular ATP quantification

The bacterial intracellular ATP was determined by ATP Assay Kit (MedChem Express USA). The log-phased *S. aureus* ATCC 43300 was collected by centrifugation at 3000 × g for 15min. The bacterial cells were washed three times and resuspended with 1× PBS to an OD630 of 0.5. Subsequently, tiliquinol was added to the suspension at a final concentration of 4 μg/mL. After incubation at 37 C for 1h, the bacterial intracellular ATP was extracted by sonication, and the supernatant was collected. The detection reagent was added to the supernatant at a 1: 9 ratio according to the manufacturer’s instructions, and the luminescence was quantified by using the microplate reader (Li et al., 2022a).

### 2.20 Competitive growth inhibition by cell wall components

Log-phased *S. aureus* was diluted with fresh TSB in the presence of 1-2×MIC of tiliquinol with 10-40 μg/mL of peptidoglycan (PGN), 128 μg/mL of N-acetylglucosamine (NAG), or 128 μg/mL of N-acetylmuramic acid (NAM). And DMSO was used as a control. The bacterial suspension was incubated at 37 °C 180 rpm at indicated time, and the growth turbidity was determined by measuring the OD630nm. Meanwhile, 5 μL of the bacterial suspension was removed to perform CFU counting at the time point of 12h (Pengfei et al., 2025).

### 2.21 Transcriptome

Log-phased *S. aureus* ATCC 43300 was treated with 10×MIC of tiliquinol at 37 °C 180 rpm for 1h with 0.1% (vol/vol) DMSO was used as a control. The bacterial cells were collected by centrifugation, and the total RNA was extracted using the TRIzol^®^ reagent (Invitrogen, USA), with the addition of lysozyme to facilitate the release of RNA. Then, rRNA was removed with Ribo-Zero Magnetic kit (Illumina, USA) and the mRAN was fragmented to 200 bp size with fragmentation buffer. The fragmented mRAN was further reverse transcribed, linked with adaptor with TruseqTM Stranded RNA sample prep Kit (Illumina, USA). The RNA-sequencing (RNA-seq) was performed with Truseq SBS kit (300 cycles, Illumina, USA) after digested with UNG enzyme (Illumina, USA) (Pisu et al., 2020). The raw data were collected and the differential expressed genes (DEGs) was further analyzed with edgeR package software. The raw data have been deposited in the NCBI (http://www.ncbi.nlm.nih.gov/bioproject/1334130) with an identifier of PRJNA1334130.

### 2.22 qRT-PCR

Log-phased *S. aureus* ATCC 43300 was cultured in TSB with or without 10×MIC of tiliquinol at 37 °C 180 rpm for 1h. Then the bacteria were harvested by centrifugation at 3000× g for 15min. The total RNA was extracted by using the E.Z.N.A. Bacterial RNA Kit (Omega, USA) according to the manufacturer’s instructions. The total RNA was reversely transcribed by using the TransScript All-in-One First-Strand cDNA Synthesis SuperMix (Transgene, Beijing, P.R. China). Quantitative PCR (qPCR) was used to quantify the mRNA levels of *uppP*, *cobQ*, *mrcA*, *femB*, *murE*, and *murF* relative to the control gene (*16S rRNA*) by using TransStart Tip Green qPCR SuperMix (Transgene, Beijing, P.R. China) (She et al., 2022). The primers used were shown in **Supplementary Table 1**.

### 2.23 Molecular docking

The structure of tiliquinol was optimized by MOPAC program. Autodock 4.2.6 And Autodock Tools 1.5.2 software were used for molecular docking assay. The docked boxes were set to wrap around the entire protein with the center point coordinates of -2.20, 99.75, and 38.22. The number of lattice points in X’Y’Z’ direction was set to “60×60×60” with a space of 0.375 Å. The number of molecular docking times was set to 100, and other parameters were set to default values. Dock tiliquinol to the active site of glucosamine-6-phosphate synthase (GlmS), phosphoglucosamine mutase (GlmM), Glucosamine-1-phosphate acetyltransfera, N-acetylglucosamine-1-phosphate uridyltransferase, UDP-N-Acetylglucosamine Enolpyruvyl Transferase (MurA), or UDP-N-acetylglucosamine-enolpyruvate reductase (MurB) proteins, respectively (Morris et al., 2009).

### 2.24 Erythrocyte lysis assay

Commercialized human red blood cells (RBCs) (Hemo Pharmaceutical and Biological Co. Shanghai, P.R. China) were diluted with 1× PBS to a final concentration of 4% (vol/vol) in the presence of indicated concentrations of tiliquinol. Meanwhile, Triton X-100 (0.1%, vol/vol) and DMSO (0.1%, vol/vol) were served as positive and negative controls, respectively. After incubation at 37 °C for 1h, the supernatant was collected by centrifugation, and the A570nm was measured (Song et al., 2021). The hemolysis rate was calculated with the following formula:


$$\text{hemolysis}\left(\%\right)=\frac{A_{Sample}-A_{0.1\text{\%DMSO}}}{A_{0.1\%\text{TritonX}-100}-A_{0.1\text{\%DMSO}}}\times 100\%$$

### 2.25 Cytotoxicity determination by cell counting Kit-8

The CCK-8 reagent (DojinDo, Japan) was used to detect the cytotoxicity of compounds against mammal cell lines of HEK293T, LO2, HepG2, HaCaT, HSF, A549, and RAW264.7. Briefly, log-phased mammal cells were diluted in 96-well plates with the optimal medium to approximately 4000 cells/well. After incubation at 37 °C with 5% CO_2_ for 24h, the unadhered cells were removed by PBS washing, and the specified concentrations of the compounds were added to each well for further 24h incubation at 37 °C. Then, 10 µL of CCK-8 was added to each well and the absorbance at 450 nm (A450nm) was recorded after 3h of incubation (Liu et al., 2015).

### 2.26 Flow cytometry

The cell apoptosis analysis was detected by using an Annexin V-fluorescein isothiocyanate (FITC)/PI (Nanjing, P.R. China) dual probes. HaCaT cells were treated with tiliquinol at the concentrations of 4 μg/mL and 32 μg/mL for 24h, respectively, followed by EDTA-free pancreatin digestion. The cells were then resuspended in 500 μL of binding buffer containing 5 μL of FITC/PI. After incubation in the dark for 10min, the samples were analyzed using a flow cytometer (BD, USA), and the apoptosis rate was calculated as reported widely (Liu et al., 2015).

### 2.27 hERG

The HEK293 cell line expressing hERG channels was cultured in DMEM medium supplemented with 10% fetal bovine serum at 37 C to approximately 70% of log phase. The cells were then washed with 1× PBS and digested with trypsin. After incubation at 37 C for 2–3 min, the cells were washed again and adjusted to a density of ~2×10^3^ cells/mL. Patch-clamp recordings were performed after the cells adhered. The extracellular fluid was prepared as 137 mM NaCl, 1 mM MgCl_2_·6H_2_O, 4 mM KCl,1.8 mM CaCl_2_·2H_2_O, 10 mM D-Glucose, 10 mM HEPES, pH=7.4. And the intracellular fluid was prepared as 140 mM KCl, 10 mM EGTA, 5 mM MgCl_2_·6H_2_O, 10 mM HEPES, 5 mM Mg-ATP, pH=7.2. The current data was collected by using Pathmaster software. And the peak current inhibition rate was calculated as:( $1-\frac{\text{Peak tail current compound}}{\text{Peak tail current vehicle}}$). The concentration-effects were fitted by the Hill equation: *I*=*I*max (1/[1+(C_1/2_/[C])^h^), where [C], C_1/2_, and h represented the drug concentration, half inhibitory concentration (IC50), and Hill coefficient, respectively (Kokot et al., 2023).

### 2.28 Human plasma protein binding

Nine hundred and eighty microliters of human plasma (IPHASE, Suzhou, P.R. China) was separately aliquoted into tubes and added with 20 μL of tiliquinol at the indicated concentrations. Warfarin sodium (0.2 μg/mL) was used as a control. Then, the mixtures were moved to the upper side of an equilibration dialysis membrane (IPHASE, Suzhou, P.R. China), and 1× PBS was added to the lower side. After incubation at room temperature for 12h, 50 μL of the solution was collected from both of the upper and lower sides, respectively. The concentration of free tiliquinol (C_f_) and total tiliquinol (C_t_) were measured with a LC-MS/MS (Triple QuadTM 5500, AB SCIEX, USA), and the protein binding rate of tiliquinol in plasma was calculated as (1-C_f_/C_t_)×100% (Zhang et al., 2025).

### 2.29 Metabolic stability in liver microsomes

For the positive control preparation, 100 μM of phenacetin was incubated with 2 μL of liver microsomes (20 mg Protein/mL, IPHASE, Suzhou, P.R. China) in the presence of 12 μL NADPH regeneration solution to a final volume of 0.2 mL. After incubation at 37 °C for 0 and 60min, respectively, the mixture was ice cooled and added with 500 μL termination solution. For the tiliquinol-treated group preparation, 100 μM of tiliquinol was incubated with 2 μL liver microsomes and 12 μL NADPH regeneration solution at 37 °C for 0, 15, 30, 45, 60 and 90min, respectively. After incubation, the concentration of tiliquinol was quantified with the LC-MS/MS as described above (Pengfei et al., 2024).

### 2.30 Abscess model

Six to seven-week-old female, specific pathogen free outbred ICR mice were anesthetized with 1% sodium pentobarbital (50 mg/kg). The back hair was shaved with an electric razor. A 50 μL of *S. aureus* ATCC 43300 suspension containing 1×10^8^ CFU/ml cells was subcutaneously injected (s.c.) into the back of the mice. The mice were randomized into three groups (N=5 mice per group): (1) Vehicle group (EL + ethanol), (2) Tiliquinol (30 mg/kg) treatment group, (3) Ampicillin (30 mg/kg) treatment group. Each group was administrated by s.c. after 1h post infection. The mice were sacrificed at 24h post infection, and their lesions were removed and homogenized for viable bacterial cells counting. Meanwhile, the lesions were dislocated and immersed in 4% paraformaldehyde for hematoxylin-eosin (H&E) staining, and immunohistochemical analysis, respectively (Zheng et al., 2024).

### 2.31 Wound infection model

Six to seven-week-old ICR female mice were selected and a wound was built by cutting approximately 6 mm-diameter skin on the back. Then 50 μL of 1×10^8^ CFU/mL bacterial suspension were dropped on the wound. One hour after the infection, 2% (wt/wt) of the tiliquinol or 2% (wt/wt) fusidic acid was applied to the infected area. After 1,3,5, and 7 days after the treatment, the wound area was measured, the viable bacterial cells in the infected skin tissues were quantified as described above. Meanwhile, the infected skins were sectioned and subjected to H&E staining for histopathological analysis (Liu et al., 2022).

### 2.32 Pharmacokinetics

Female ICR mice aged 6–7 weeks were administered subcutaneously (s.c.), intraperitoneally (i.p.) or orally (p.o.) with tiliquinol at the dosage of 30 mg/kg. In addition, the intravenously (i.v.) injected mice were administered with 10 mg/kg of tiliquinol. Blood samples were collected from the jugular vein at the time points of 0.083, 0.25, 0.5, 1, 2, 4, 8, and 24h post-treatment. Plasma was separated by centrifugation at 6,800×g for 6min. The compounds concentrations in the plasma were quantified by using a LC-MS/MS-04 instrument (TQ4000, JEOL, Japan), and the pharmacokinetic parameters were calculated by using Phoenix WinNonlin 7.0 software (Certara, Princeton, NJ, USA) (Ekins et al., 2018).

### 2.33 Peritonitis-sepsis model

Female, 6-8-week-old ICR mice were obtained from Hunan SJA Experimental Animal Co. Ltd. (Changsha, P.R. China). Tiliquinol was dissolved in a Kolliphor/ethanol mixture (1:1, vol/vol) and then diluted 1:10 in saline. The overnight cultured *S. aureus* ATCC 43300 was washed twice with saline and adjusted to 1 McF. Each mouse was i.p. injected with 500 μL of the bacterial suspension containing 5% (wt/vol) mucin (Cool Chemistry, Beijing, P.R. China). At 1h post-infection, the mice (n = 10/group) were administered with 30 mg/kg of tiliquinol. Saline, containing 5% Kolliphor with 5% ethanol, was served as the vehicle group. After 24h post the treatment, the mice were sacrificed, and the livers, spleens, lungs, and kidneys were collected for viable bacterial enumeration (Pengfei et al., 2022).

### 2.34 Periprosthetic joint infection model

Six-week-old female ICR mice were randomly divided into 4 groups: (1) Sham group without infection or drug treatment. (2) Vehicle group (the infected mice treated with 5% Kolliphor with 5% ethanol). (3) Infected mice treated with 30 mg/kg of tiliquinol. (4) Infected mice treated with 30 mg/kg of vancomycin. *S. aureus* ATCC 43300 was overnight cultured at 37 °C 180 rpm, washed and resuspended in saline. Mice were anesthetized with 1% (wt/vol) pentobarbital sodium. Their knees were disinfected with 75% ethanol, and then dissected, exposed the femur. A 0.3 mm×0.8 mm needle (Sinocare, Changsha, P.R. China) was inserted into the femur as an implant. The skin was sutured and injected with 1×10^6^ CFU *S. aureus*. Then, the mice were administrated consecutively with the indicated reagents for 7 days by i.p. injection as described above in each group. At 1, 3, 5, and 7 days post infection, the bacterial burden of joint bone tissue and surrounding soft tissue were counted by CFU enumeration. Meanwhile, at 21 days after infection, the knees with implants were collected for micro-CT scanning (Bruker SkyScan1276, Bruker Micro-CT, Germany). The parameters of bone mineral density (BMD), bone volume/total volume (BV/TV), trabecular thickness (TB.TH), trabecular number (TB.N), and trabecular Separation/Spacing (TB.SP) of each group were analyzed by the CTAn program (version 1.18.4.0, Skyscan Company, Bruker Micro-CT, Germany). In addition, the femurs of the mice were harvested, fixed with 4% paraformaldehyde, and performed with pathological examination (Jiang et al., 2023).

### 2.35 *In vivo* toxicity

Two groups of mice (n = 5/group) were i.p. injected with 5% Kolliphor + 5% ethanol or 30 mg/kg of tiliquinol, respectively. After 24h post the treatment, blood samples were collected to quantitative determination of hematological parameters and organic biomarkers. Meanwhile, the hearts, livers, spleens, lungs and kidneys of the mice were removed and preserved in 4% paraformaldehyde for H&E staining (Liu et al., 2021).

### 2.36 Statistical analysis

Data were analyzed with GraphPad Prism 9.0 software (GraphPad Software Inc., CA, USA). Comparisons between two independent groups were conducted using Student’s t-test, one-way ANOVA and Dunnett’s multiple comparisons test were applied for comparisons among multiple groups. Data are presented as mean ± standard deviation (SD) from at least three independent experiments. Statistical significance was considered with *P*<0.05.

## 3 Results

### 3.1 Bactericidal activity of tiliquinol against *S. aureus*

**Figure 1:**
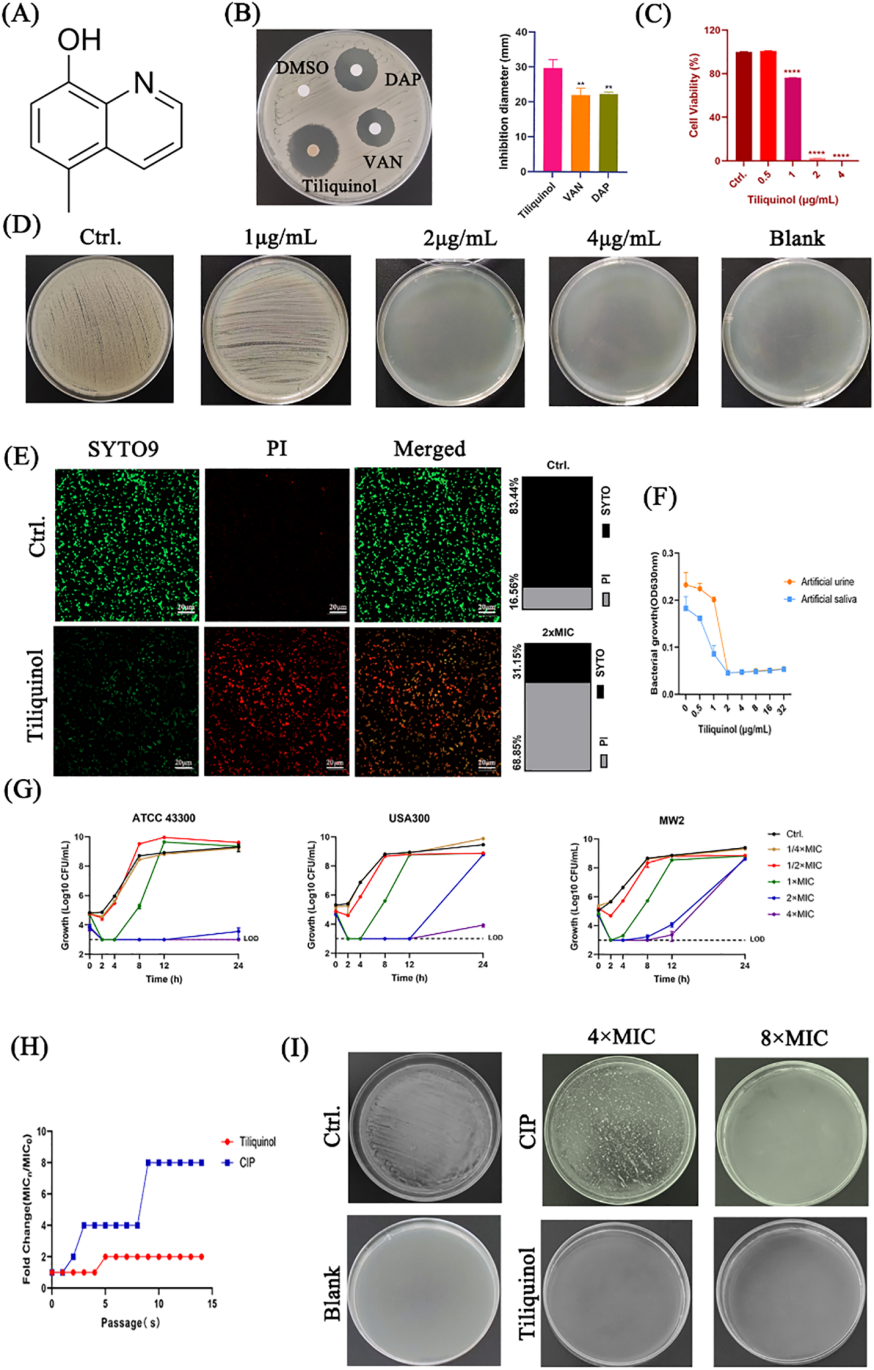
Antimicrobial activity and resistance frequency of tiliquinol against MRSA ATCC 43300. **(A)** The chemical structure of tiliquinol. **(B)** The K-B test was used to determine the drug sensitivity of tiliquinol, with DMSO as the negative control and DAP and VAN as the positive controls (left). The amount of each drug was 100 µg. Quantification of the size of the inhibition zone (right). **(C)** XTT reduction assay was employed to determine the cell viability of planktonic bacteria following treatment with various concentrations of tiliquinol. **(D)** Representative images depicting *S. aureus* ATCC 43300 treated with various concentrations of tiliquinol. **(E)** Following a 2 h treatment with tiliquinol at a concentration of 1×MIC, the ratio of live cells to dead cells was assayed using SYTO9/PI staining. Scale: 20 µm. **(F)** The MIC of tiliquinol in artificial urine and saliva. **(G)** The time-kill curve of tiliquinol against *S. aureus*. **(H)** The capacity of tiliquinol to induce resistance in *S. aureus* ATCC 43300 at a sub-MIC (1/2×MIC) was investigated. Ciprofloxacin (CIP) was employed as the positive control. **(I)** Representative images depicting one-step drug resistance following treatment with tiliquinol. ** p < 0.01; **** p < 0.0001.

Tiliquinol is an aromatic compound characterized by a quinoline ring and a phenolic hydroxyl group in its molecular structure (**Figure 1A**). It was found to be effective against both type strains and clinical isolates of methicillin-sensitive *S. aureus* (MSSA) and MRSA, with MIC values of 2 μg/mL while MBC values ranging from 2 to 4 μg/mL. Additionally, tiliquinol also demonstrated bactericidal activity against *S. epidermidis* and *Enterococcus*, with the MIC and MBC values of 1-4 μg/mL and 1-16 μg/mL, respectively. However, no antimicrobial activity against Gram-negative bacteria was observed, as the MIC values exceeded 16 μg/mL (**Table 1**). The Kirby-Bauer test revealed that the inhibition zone of tiliquinol was larger than that observed for VAN and daptomycin at the same dosage, exhibiting a clear boundary with diameters ranging from 27–32 mm (**Figure 1B**). Furthermore, treatment with sub-MIC concentration of tiliquinol could significantly reduce the viability of MRSA ATCC 43300 cells (**Figure 1C**). On MH plates, no growth of ATCC 43300 was detected following treatment with the 1×MIC (2 μg/mL) of tiliquinol indicating strong bactericidal activity by tiliquinol (**Figure 1D**). Bacterial live/dead cells detection by SYTO9/PI fluorescent probes indicated a significant increase in damaged or dead cells among ATCC 43300 after treated with 2×MIC of tiliquinol for 2h (**Figure 1E**). Notably, even in artificial urine and saliva environments, tiliquinol still exhibited significant antimicrobial activity against *S. aureus* with the MIC of 2 μg/mL (**Figure 1F**). The time-killing curve exhibited that treatment with 1×MIC of tiliquinol rapidly reduced the viable cell counts of all tested strains to the limit of detection within 2h in a concentration-dependent manner (**Figure 1G**). Multi-step resistance-inducing assay indicated that consecutive exposure to sub-MIC concentrations of tiliquinol over 15 days did not result in resistance mutations of *S. aureus*, in contrast, the MIC value for ciprofloxacin (CIP) was 8-fold increased (**Figure 1H**). And the last generation of CIP-induced highly resistant *S. aureus* still remained susceptible to tiliquinol with the MIC of 4 μg/mL, which indicated none cross-resistance occurred. Similarly, by one-step resistance-inducing assay, no resistant strains was emerged in the treatment groups exposed to 4-8×MIC of tiliquinol, while resistant colonies observed by the treatment of 4×MIC of CIP (**Figure 1I**). In addition, tiliquinol exhibited a rapid bactericidal effect by post-antibiotic effect test (PAE) (**Supplementary Figure 1**).

**Table 1:** Antimicrobial susceptibility test against ESKAPE.

Strains	Characteristics	Tiliquinol		TI
		MIC	MBC	
*S. aureus*				
ATCC 43300	Type strain, MRSA	2	2	83.35
USA300	Type strain, MRSA	2	2	83.35
LZB1	Clinical isolate, MSSA, BF	2	4	83.35
ATCC 29213	Type strain, MSSA	2	4	83.35
ATCC 25923	Type strain, MSSA	2	2	83.35
MW2	Type strain, MSSA	2	2	83.35
RN4220	Type strain, MSSA	2	2	83.35
SAJ1	Clinical isolate, MRSA, VISA	2	2	83.35
RJ-2	Clinical isolate, MSSA	2	4	83.35
80416	Clinical isolate, MRSA	2	4	83.35
82174	Clinical isolate, MRSA	2	4	83.35
82231	Clinical isolate, MRSA	2	2	83.35
*S. epidermidis*				
RP62A	Type strain, MSSE, BF	1	1	166.7
ATCC 12228	Type strain, MSSE, NBF	1	1	166.7
S1	Clinical isolate, MSSE	2	2	83.35
S3	Clinical isolate, MSSE	1	2	166.7
S2	Clinical isolate, MSSE	2	2	83.35
S11	Clinical isolate, MSSE	2	2	83.35
*E. faecalis*				
ATCC 29212	Type strain, VSE	2	2	83.35
ATCC 51299	Type strain, VRE	2	2	83.35
*E. faecium*				
ATCC 19434	Type strain, VSE	2	2	83.35
U101	Clinical isolate, VRE	1	1	166.7
SRYEFM1	Clinical isolate, VRE	2	2	83.35
SRYEFM13	Clinical isolate, VRE	4	16	41.68
SRYEFM2	Clinical isolate, VRE	1	1	166.7
SRYEFM5	Clinical isolate, VRE	1	1	166.7
SRYEFM9	Clinical isolate, VRE	2	4	83.35
SRYEFM6	Clinical isolate, VRE	2	2	83.35
SRYEFM15	Clinical isolate, VRE	4	4	41.68
*E. coli*				
ATCC 25922	Type strain	32	>32	5.21
*K. pneumoniae*				
ATCC 700603	Type strain	32	>32	5.21
*A. baumannii*				
ATCC 19606	Type strain	16	>32	10.42
*P. aeruginosa*				
PAO1	Type strain	>32	>32	>5.21

MIC(μg/mL), MBC(μg/mL). VSE, vancomycin-susceptible *enterococci*; VRE, vancomycin-resistant *enterococci*; VISA, Vancomycin Intermediate *S. aureus*; NBF, biofilm formation negative; BF, biofilm formation positive.

### 3.2 Effective anti-biofilm effect of tiliquinol

**Figure 2:**
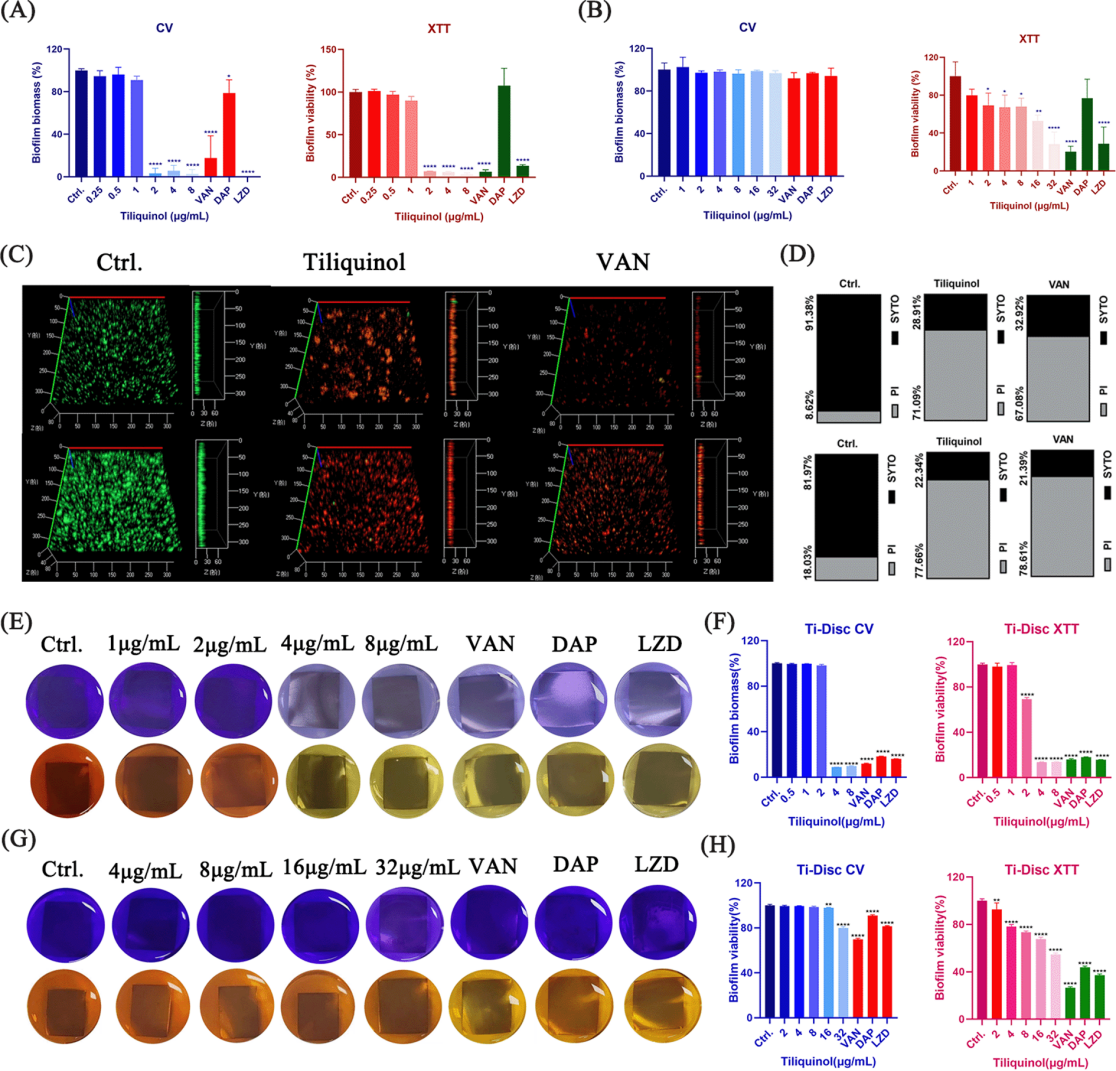
Anti-biofilm effect of tiliquinol against MRSA ATCC 43300. **(A)** The biofilm inhibitory activity of tiliquinol against *S. aureus* was measured using crystal violet staining (left) and XTT reduction assay (right), respectively. **(B)** The eradication efficacy of tiliquinol against the pre-formed biofilm of *S. aureus* was evaluated using crystal violet staining (left) and XTT reduction assay (right), respectively. **(C)** Observation of the biofilm inhibitory effect of tiliquinol by SYTO9/PI staining (up). Evaluation of the biofilm eradication activity of tiliquinol by SYTO9/PI staining (down). SYTO9 and PI were employed to track live cells and dead cells, respectively. **(D)** Quantitative analysis for panel **(C)** The inhibitory effect **(F)** and eradication effect **(H)** of tiliquinol on *S. aureus* biofilm formed on Ti discs were measured using crystal violet staining (up) and XTT reduction assay (down), respectively. Representative images of the inhibitory **(E)** and eradicating **(G)** effects are also presented. * p < 0.05; ** p < 0.01; **** p < 0.0001.

The formation of biofilms can lead to serious infections in the human body and the anti-biofilm effects by tiliquinol was thoroughly assessed in the present study. As shown in **Figure 2A**, like conventional antibiotics of Linezolid (LZD) and VAN, tiliquinol exhibited significant biofilm inhibition activity for total biomass and viability at the concentration of 2 μg/mL, as determined by crystal violet staining and XTT reduction assay, respectively (**Figure 2A**). Although, the pre-formed biofilm biomass showed resistant to all the antimicrobials as determined by crystal violet staining, tiliquinol could significantly reduce its viability at the concentration of 2 μg/mL and in a concentration-dependent manner (**Figure 2B**). In addition, an anti-biofilm effect of tiliquinol was also observed in *S. epidermidis* (**Supplementary Figure 2**). As observed by CLSM, treatment with tiliquinol at the concentration of 2 μg/mL significantly inhibited the biofilm formation, while 8 μg/mL of tiliquinol could effectively eradicate pre-formed biofilms by *S. aureus.* (**Figure 2C, D**). Next, the antibiofilm activity by tiliquinol against *S. aureus* on titanium (Ti) discs surfaces was also determined. As shown in **Figure 2E**, for biofilm inhibition, substantial reductions in both biofilm biomass and cell viability were observed after treated with tiliquinol (**Figure 2F**). Furthermore, the significant eradication of pre-formed biofilms on the Ti-discs was also observed after treated with tiliquinol (**Figure 2G**). Consistently, quantitative analysis demonstrated significantly reduced cell viability within the biofilms after the treatment (**Figure 2H**).

### 3.3 Tiliquinol disrupts ATP synthesis by interacted with PMF

**Figure 3:**
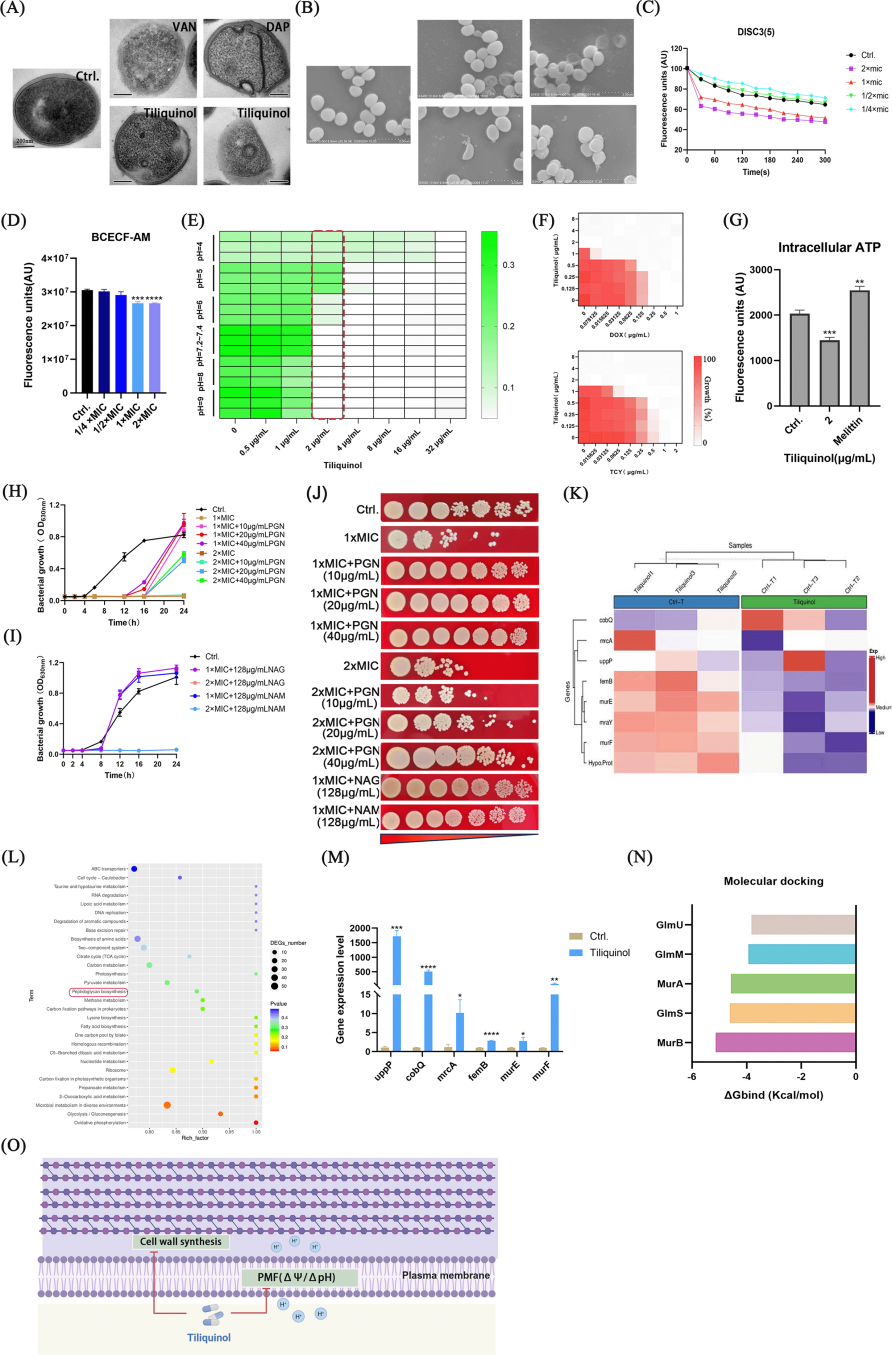
Mechanism of action. **(A)** Observation of the ultrastructure of *S. aureus* by TEM after treatment with tiliquinol for 1 h. Scale: 200 nm. **(B)** Observation of the ultrastructure of *S. aureus* by SEM after treatment with tiliquinol for 1 h. Scale: 200 µm. **(C)** Tiliquinol altered the fluorescence intensity of the DiSC3(5) probe. **(D)** The intracellular pH of *S. aureus* was measured using the BCECF-AM probe. **(E)** Determination of the MIC of tiliquinol under different pH conditions. **(F)** The checkerboard assay was employed to assess the combinatorial use of tiliquinol with doxycycline (DOX) and tetracycline (TCY). **(G)** Intracellular ATP quantification. **(H)** Competitive growth inhibition curves of tiliquinol in combination with peptidoglycan. **(I)** Competitive growth inhibition curves of tiliquinol in combination with cell wall components of NAG and NAM. **(J)** Representative images of the competitive growth inhibition of tiliquinol in combination with peptidoglycan, NAG, and NAM. **(K)** Gene Ontology analysis of the differential expression of cell-wall associated genes identified through whole transcriptome sequencing **(L)** KEGG enrichment analysis of DEGs in *S. aureus* following treatment with tiliquinol. **(M)** Determination of expression levels of cell wall-related genes by qRT-PCR. **(N)** The binding energies between tiliquinol and potential targets of cell-wall-associated enzymes by molecular docking. **(O)** Mechanistic hypothesis. Tiliquinol demonstrates antibacterial activity against *S. aureus* primarily by disrupting the PMF and inhibiting cell wall. * p < 0.05; ** p < 0.01; *** p < 0.001; **** p < 0.0001.

As observed by transmission electron microscopy (TEM), treatment of MRSA ATCC 43300 with 10×MIC tiliquinol for 30min resulted in disruption of cell integrity and uneven density of intracellular contents (**Figure 3A**). Similarly, scanning electron microscopy (SEM) also indicated that the cells treated with tiliquinol exhibited leakage of cytoplasmic contents and morphological atrophy (**Figure 3B**). It was reasonably speculated that the permeability of the membrane or collapse of the PMF may be altered by tiliquinol. However, none increase of the membrane permeability was determined after treated with tiliquinol by SYTOX Green fluorescent probe monitoring (**Supplementary Figure 3**). PMF is an electrochemical gradient on both sides of the bacterial cell membrane that drives many basic physiological processes, including biofilm formation, ATP synthesis, nutrient uptake, and waste excretion. We found that tiliquinol exhibited PMF disruption activity against MRSA as determined by DiSC3(5) probe (**Figure 3C**). And a decreased fluorescence intensity of the BCECF-AM probe indicated the ΔΨ, a component of PMF, disruption activity by tiliquinol (**Figure 3D**). As we expected, the antimicrobial activities of tiliquinol were changed in the presence of varying proton concentrations (**Figure 3E**). Tiliquinol disrupted the PMF and cell wall structure of *Staphylococcus aureus*, thereby inhibiting the bacterium’s ability to mount an effective resistance response against tetracycline (TCY). By the checkerboard assay, a partial synergy effect was discovered between tiliquinol and tetracyclines (**Figure 3F**). Due to PMF serves as a driving force for ATP synthesis, its disruption could potentially trigger remodeling of the electron transport chain (Yang et al., 2023). As we expected, after treated *S. aureus* with 2 μg/mL of tiliquinol, a decrease in intracellular ATP levels was observed (**Figure 3G**).

### 3.4 Tiliquinol inhibited peptidoglycan synthesis

Since the disruption of cellular integrity was observed by electron microscopy, we supposed that destroying the cell walls of bacteria might be another potential target of tiliquinol. As we expected, the growth inhibitory activity of tiliquinol was obviously inhibited in the presence of exogenous peptidoglycan (**Figure 3H**). Further, we observed that both exogenous NAG and N-acetylmuramic acid (NAM), main components of peptidoglycan, could effectively hinder the antibacterial efficacy of tiliquinol (**Figure 3I**). Consistently, the viable cell counts obtained from the assays also indicated the competitive inhibitory activity of these cell wall components against tiliquinol (**Figure 3J**). Next, transcriptome analysis indicated that the genes associated with peptidoglycan biosynthesis (**Figure 3K**) or crosslinking pathways (including *cobQ*, *mrcA*, *uppP*, *femB*, *murE*, *mraY*, *murF*, and *Hypo. Prot*) exhibited significant differential expression in response to tiliquinol treatment (**Figure 3L**). In consistence, qRT-PCR also confirmed these DEGs (**Figure 3M**). To further explore the interaction between tiliquinol and targets, molecular docking was performed between tiliquinol and the key enzymes involved in NAD or NAM synthesis. These enzymes were tightly bound to tiliquinol(**Figure 3N**; **Supplementary Figure S4**). Collectively, these results indicated that tiliquinol inhibits peptidoglycan biosynthesis, thereby damaging the integrity and stability of the cell wall.

### 3.5 Acceptable toxicity of tiliquinol

**Figure 4:**
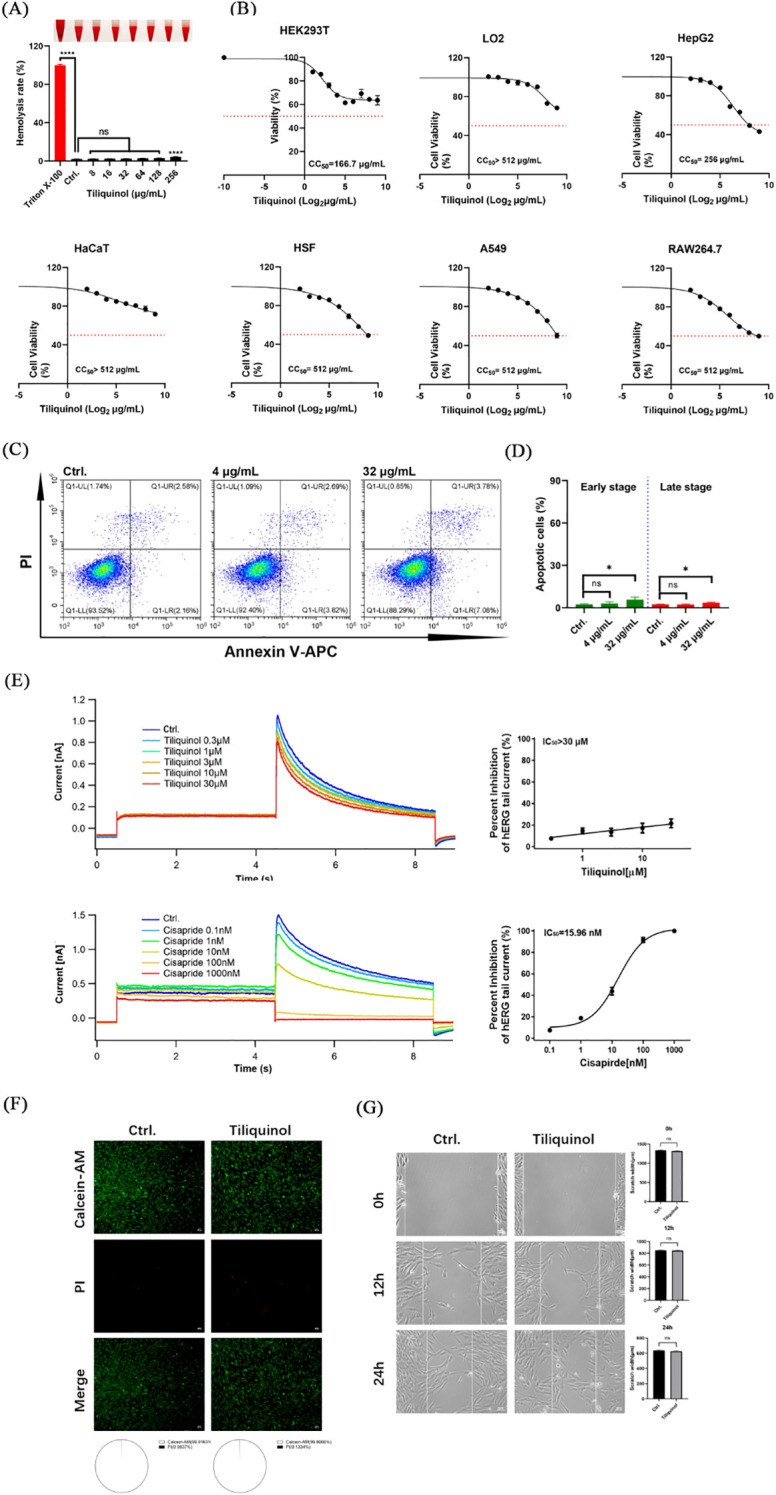
Cytotoxicity profile of tiliquinol. **(A)** Erythrocytes were incubated with tiliquinol at designated concentrations ranging from 0 to 256 μg/mL. DMSO (0.1%) and Triton X-100 (0.1%) were employed as the negative and positive controls, respectively. **(B)** Cell viability of HEK293T, LO2, HepG2, HaCaT, HSF, A549, and RAW264.7 cell lines following treatment with tiliquinol. CC_50_ represents the half-maximal cytotoxic concentration. **(C)** The apoptosis of cells induced by tiliquinol at concentrations of 4 μg/mL and 32 μg/mL was analyzed by flow cytometry. **(D)** Apoptosis rate quantification for panel **(C) (E)** The cardiotoxicity of tiliquinol was evaluated using the hERG assay. Cisapride served as the positive control. **(F)** CLSM images of calcein-AM/PI staining in HSF cells following treatment with tiliquinol. Live cells were stained green, while dead cells were stained red. Scale: 100 μm. **(G)** Representative images of the scratch wound healing assay using HSF cells treated with 4 μg/mL tiliquinol. Scale: 100 μm.

No hemolysis of human red blood cells by tiliquinol was detected even at the concentration up to 128 μg/mL (**Figure 4A**). Tiliquinol also exhibited extremely low cytotoxicity to human cell lines of HEK293T, LO2, HepG2, HaCaT, HSF and A549 as well as the mouse monocyte macrophage RAW264.7 with the IC_50_ values above 128 μg/mL (**Figure 4B**). Meanwhile, no significant difference in the apoptosis rate of HaCaT between the control group and those treated with either 2×MIC or 16×MIC tiliquinol was observed after 24h treatment (**Figure 4C, D**). The hERG assay indicated that tiliquinol did not exhibited inhibitory activity against potassium channels with the IC_50_ exceeded 30 μM while the positive control (cisapride) demonstrated a significant inhibition (**Figure 4E**). Considering that skin and soft tissue infections are primarily caused by *S. aureus* (Pengfei et al., 2024), we further investigated the toxic effects of tiliquinol on human skin fibroblasts cell line HSF by using Calcein-AM/PI staining. As shown in **Figure 4F**, almost no cell damage was observed following treatment with 4 μg/mL tiliquinol. Additionally, treatment with 2×MIC tiliquinol exhibited none impact on the migration ability of HSF as compared to the control group (**Figure 4G**). Next, the acute *in vivo* toxicity was evaluated by administration (i.p.) with tiliquinol at the dosage of 30 mg/kg. The results indicated no significant differences in the liver biomarker glutamic-pyruvic transaminase (ALT; **Supplementary Figure 6A**), the kidney biomarker blood urea nitrogen (**Supplementary Figure 6B**), the platelet counts (**Supplementary Figure 6C**), the white leukocyte parameters (**Supplementary Figure 6D**), or the erythrocyte parameters (**Supplementary Figure 6E**). Besides, none pathological changes were observed in the main organs of heart, liver, spleen, lungs, or kidneys as assessed by hematoxylin-eosin (H&E) staining (**Supplementary Figure 6F**).

### 3.6 *In vivo* antimicrobial efficacy by tiliquinol

**Figure 5:**
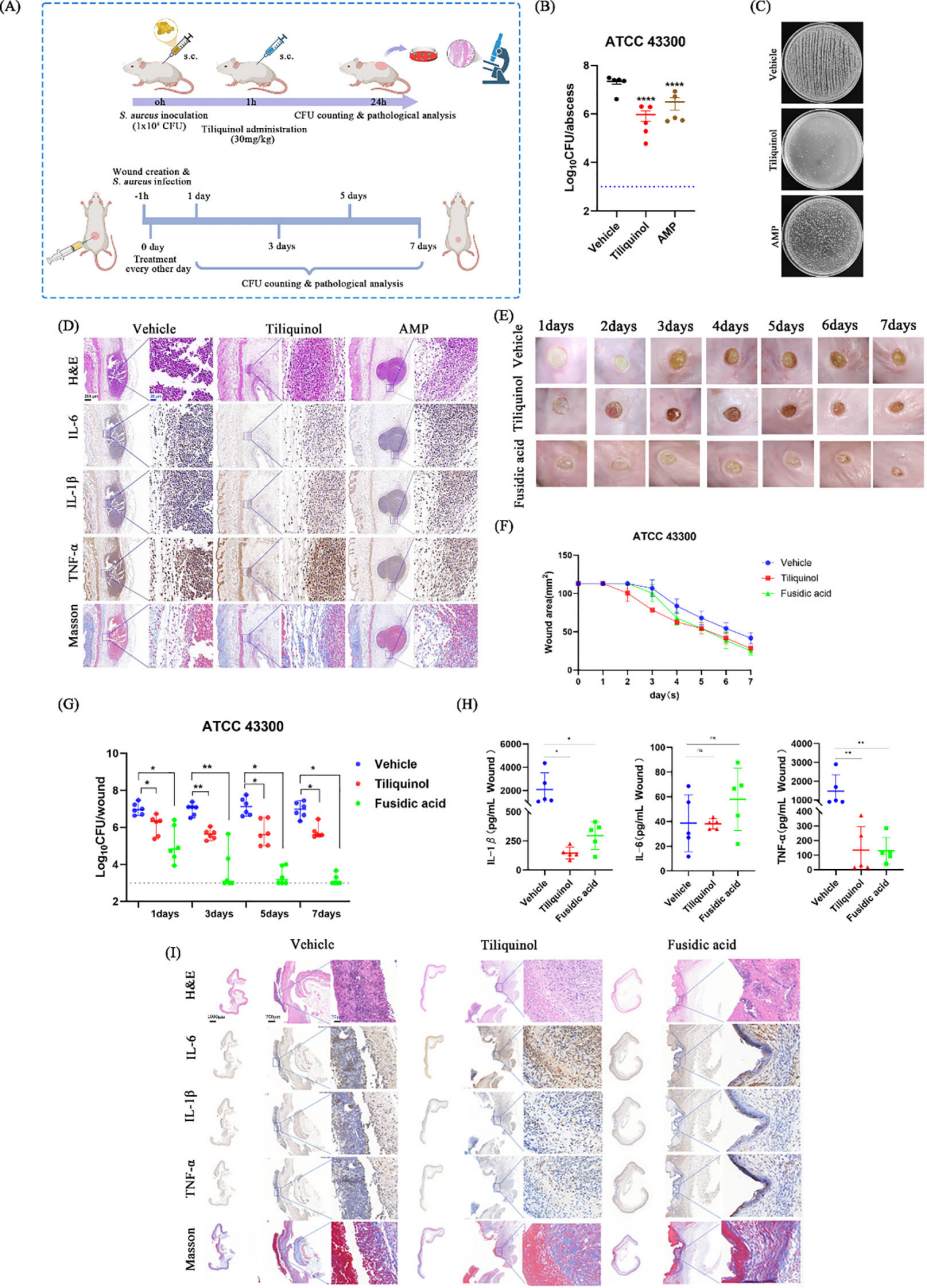
*In vivo* antibacterial effect of tiliquinol against MRSA ATCC 43300 in Skin and soft tissue infection model. **(A)** Schematic illustration of the preparation of abscess and wound infection model. **(B)** Viable cell counts in abscesses. **(C)** Representative images for the counting of live cells in abscesses. **(D)** Pathological examination of abscesses was performed by H&E staining and immunohistochemistry (IL-6, IL-1β, TNF-α, and Masson), respectively. **(E)** Representative images of treated and untreated wounds. **(F)** Curve of wound area over time. **(G)** Viable cell counts of wounds at 1, 3, 5, and 7 days after the treatment. **(H)** Quantification of cytokines (IL-6, IL-1β, and TNF-α). **(I)** Pathological detection of the infected wounds by H&E staining and immunohistochemistry. * p < 0.05; ** p < 0.01; **** p < 0.0001.

Firstly, the *in vivo* antibacterial activity of tiliquinol was investigated by using subcutaneous abscesses and wound infections models, respectively (**Figure 5A**). It was observed that tiliquinol significantly reduced the bacterial load in abscesses (**Figures 5B, C**). Histological examination through H&E staining and immunohistochemical analysis revealed that pronounced abscess formation in the vehicle or AMP-treated group, characterized by extensive infiltration of inflammatory cells and elevated expression levels of inflammatory factors such as IL-6, IL-1β, and TNF-α. However, following treatment with tiliquinol, both the area of the abscess and levels of inflammatory factors were significantly reduced, while the collagenousous fiber was increased indicating the tissue repairing (**Figure 5D**). Similarly, the wound infection model also demonstrated the potential antibacterial efficacy by tiliquinol. As shown in **Figure 5E** and F, the wound areas were obviously decreased after treatment with tiliquinol for a consecutive 7 days. And tiliquinol could also significantly reduce the bacterial loads in the wounds at indicated time points (**Figure 5G**). In addition, there was a marked reduction in the expression levels of inflammatory factors of IL-1β and TNF-α (**Figure 5H**), which was consistent with the results of H&E staining and immunohistochemical analysis (**Figure 5I**).

**Figure 6:**
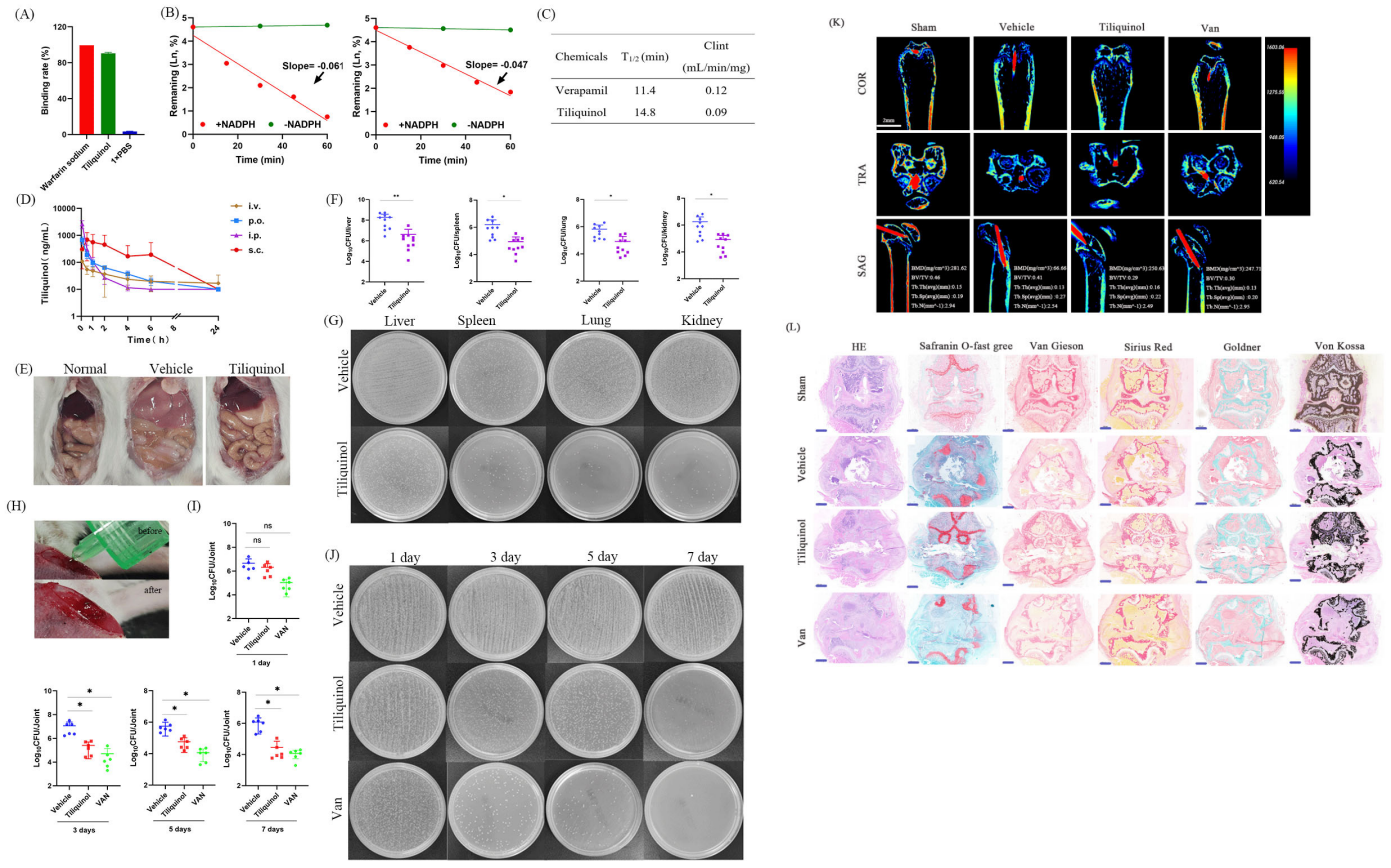
*In vivo* antibacterial effect of tiliquinol against MRSA ATCC 43300 in peritonitis-sepsis model and Periprosthetic joint infection (PJI) model. **(A)** Plasma protein binding ratio by tiliquinol. Warfarin was used as a positive control. **(B)** Enzyme kinetics of tiliquinol metabolism in the presence of mice liver microsomes. **(C)** Half-life and clearance rate calculation. **(D)** Pharmacokinetic curves of tiliquinol. **(E)** Representative images of normal mice and peritonitis model mice without treatment or after treatment. **(F)** Tiliquinol effectively reduced the viable bacterial cell loads in mice organs. **(G)** The CFU count showed a representative image of the antibacterial effect after treatment with 30 mg/kg tiliquinol in the peritonitis-sepsis model. **(H)** Representative images of Ti needle implantation. **(I)** The viable cells count of the infected joints after treated with tiliquinol for 1 day, 3 days, 5 days, and 7 days. VAN was the positive control. **(J)** The CFU count showed a representative image of the antibacterial effect after treatment with 30 mg/kg tiliquinol in the PJI model. **(K)** Bone mineral density heat-map and parameters of the infected joints 21 days postinfection. COR, coronal. TRA, transverse. SAG, 3D reconstruction and sagittal. **(L)** Pathological analysis of the implants and surrounding tissues at day 21. Scale: 500 µm. ns = not significant; * p < 0.05; ** p < 0.01.

Tiliquinol exhibited a high plasma protein binding ability (**Figure 6A**), while remained relatively unstable in the presence of liver microsomes with the *T_1/2_* of 14.8min (**Figures 6B, C**). The pharmacokinetic curve indicated the half-live of tiliquinol ranged from 0.43 to 3.96h across different administration routes. It was noted that the antimicrobial effects of tiliquinol could be quickly reached with low *T*_max_ ranged from 0.08 to 0.81h. And the i.p. injection could achieve the optimal *C*_max_ of 2.53 μg/mL, which exceeded the MIC value. Although, the *C*_max_ values were dissatisfied through other administration routes, significant accumulation in specific tissues is considered possible. In addition, the bioavailability of tiliquinol by p.o., i.p., and s.c. administration were 48.27% (48.27 ± 10.45), 68.89% (68.89 ± 23.16), and 91.97% (91.97 ± 58.13), respectively (**Figure 6D**; **Table 2**). Based on these findings, further *in vivo* antibacterial activity of tiliquinol in systemic infection models was conducted by i.p. injection. The *in vivo* systemic antibacterial activity of tiliquinol was further investigated by using a peritonitis-sepsis model. As shown in **Figure 6E**, the edema of the infected liver was obviously reduced after treated with tiliquinol. In consistence, the bacterial loads in the liver, spleen, lung, and kidney were also decreased in the tiliquinol treatment group (**Figures 6F, G**). Given its potent antibiofilm ability, the *in vivo* efficacy of tiliquinol was further evaluated by using a periprosthetic joint infection (PJI) model (**Figure 6H**). Similar as VAN, tiliquinol could also effectively reduce the bacterial loads of the infected joints on day 3, 5 and 7, respectively, as determined by CFU counting (**Figures 6I, J**).

**Table 2:** The pharmacokinetic parameters of tiliquinol.

Route	mg/kg	T1/2 (h)	Tmax (h)	Cmax (μg/L)	AUC (0-t)	AUC (0-∞)	MRT (0-t)	MRT (0-∞)	F (%)
					h*μg/L	h*μg/L	h	h	
i.v.	10	3.49 ± 3.58	0.66 ± 0.73	188.21 ± 164.41	342.91 ± 182.45	578.07 ± 487.57	3.65 ± 4.85	7.76 ± 10.30	‐
p.o.	30	1.93 ± 0.37	0.08 ± 0.00	675.78 ± 1.97	496.59 ± 107.54	546.33 ± 112.16	1.51 ± 0.17	2.32 ± 0.56	48.27 ± 10.45
i.p.	30	0.43 ± 0.14	0.08 ± 0.00	2528.20 ± 9.30	708.66 ± 238.28	714.80 ± 244.65	0.44 ± 0.11	0.49 ± 0.13	68.89 ± 23.16
s.c.	30	3.96 ± 5.19	0.81 ± 0.80	502.50 ± 416.60	946.16 ± 644.95	1246.97 ± 5597.97	2.01 ± 0.70	8.03 ± 11.28	91.97 ± 58.13

Subsequently, imaging changes of the infected joints were assessed by micro-CT. As illustrated in **Figure 6K**, the images confirmed successful implantation of all insulin needles in the femur from the knee joint. Meanwhile, three-dimensional reconstruction revealed that, compared with the vehicle group, the joint surfaces in the tiliquinol group exhibited more favorable healing with higher bone density. Consistently, quantitative analyses of the bone mineral density, trabecular thickness, and trabecular separation/spacing also displayed an improved bone density increasement and trabecular recovery. Next, the pathological analysis of the PJI model was conducted. As shown in **Figure 6L**, H&E staining revealed that obvious structural damage and extensive inflammatory cells infiltration of the infected joints were observed in the vehicle group, while, the morphology of the joints was remained relatively intact in the tiliquinol or VAN-treated groups. Safranin O-fast green staining indicated that severe damage of cartilage was detected in the vehicle group, while an alleviation in the tiliquinol treatment group was observed. Similarly, Van Gieson staining also demonstrated an abundance of well-stained collagenous fibers presented in the sham group or tiliquinol treatment group. Further, compared with the vehicle group, Sirius Red staining confirmed an increasement of collagenous fiber in the tiliquinol treatment group, and Goldner staining indicated enhanced proportion of mineralized bone in the tiliquinol treated group. The Von Kossa staining indicated that the bone healing was enhanced in the tiliquinol treated group with increased calcium salt deposition.

### 3.7 Structure-related activity

**Figure 7:**
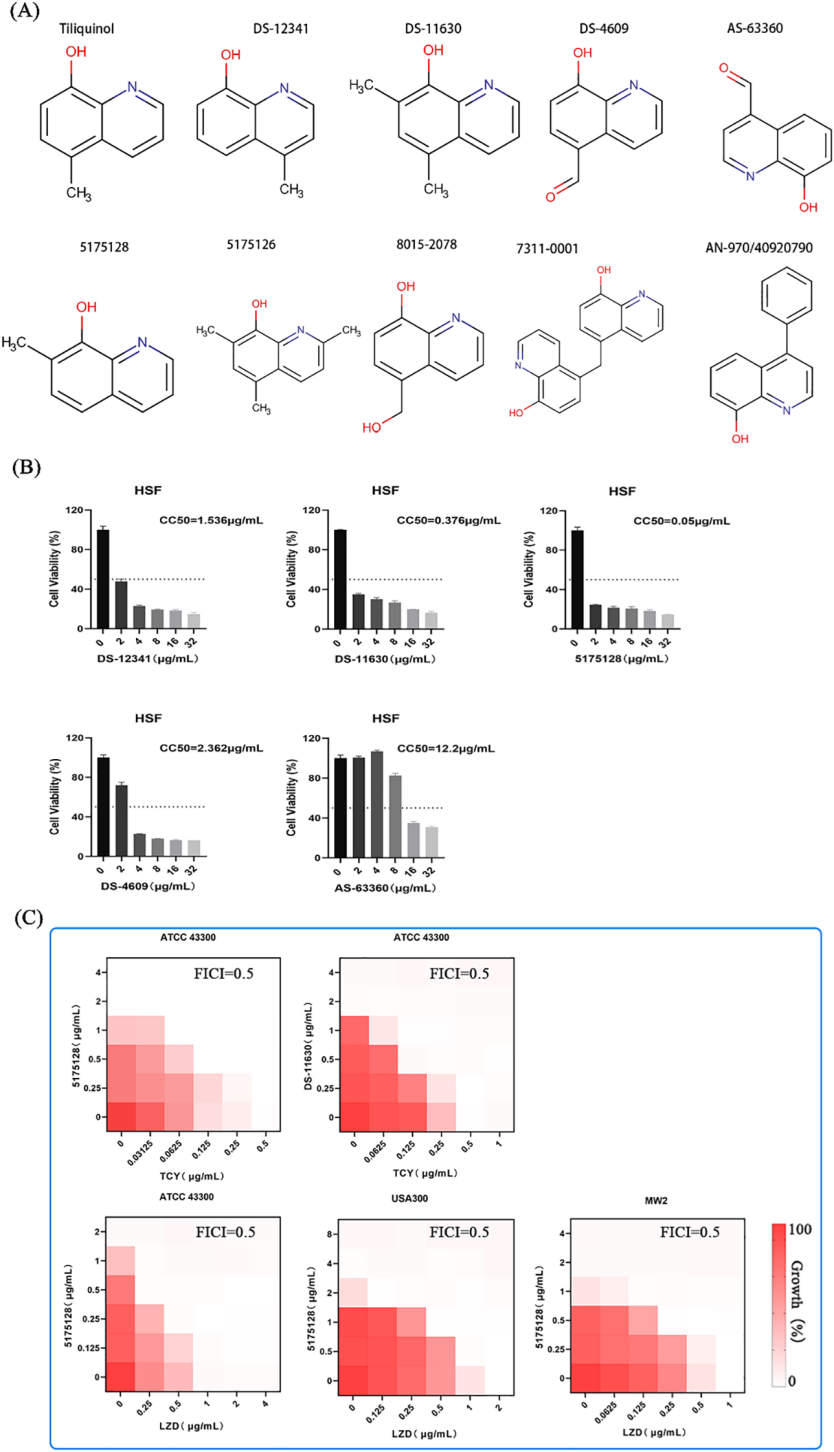
Analogues of tiliquinol. **(A)** The molecular structural formulas. **(B)** Cell viability of HSF cell lines treated with the selected 5 analogues. CC_50_, related 50% inhibitory concentration. **(C)** The synergistic antibacterial activity against *S. aureus* type strains ATCC 43300 was observed between 5175128 and TCY, as well as between DS-11630 and TCY. 5175128 and LZD had synergistic activity against different *S. aureus* type strains.

The core scaffold of tiliquinol is identified as a quinoline ring, which consists of a benzene ring fused with a pyridine ring. On the benzene moiety of tiliquinol, a hydroxyl group and a methyl substituent are present. By searching the ZINC15 database with the structure similarity ≥80%, 9 derivatives featuring quinoline rings as the primary scaffold were investigated for their antibacterial activities against *S. aureus* (**Figure 7A**). It was observed that the MIC values of 5 derivatives ranged from 1-16 μg/mL, in which DS-12341 exhibited the most potent antibacterial activity among them with the MIC of 1 μg/mL. As observed, the methyl group was repositioned from the 5-position to the 4-position on the quinoline ring of the tiliquinol to result the formation of DS-12341. Additionally, the addition (DS-11630) or shift (5175128) of methyl group on the benzene ring of tiliquinol demonstrated unchanged antimicrobial activities but higher cytotoxicity (**Figure 7B**; **Table 3**). Notably, a significant increase in MIC values was observed for the compounds DS-4609 and AS-63360 following substitution of methyl groups with formyl groups on both benzene ring or pyridine ring. In addition, the MICs of other derivatives were detected to be more than 32 μg/mL. Next, we evaluated the cytotoxicity of the derivatives with MIC values less than 32 μg/mL. However, the CC_50_ and therapeutic indexes of these derivatives were all found to be lower than tiliquinol (**Figure 7B**,;**Table 3**). These findings suggest that the tiliquinol itself could be the most optimal structure among derivatives with the highest antimicrobial activity while lowest cytotoxicity.

**Table 3:** Antimicrobial activity of tiliquinol analogs against *S. aureus* ATCC 43300.

Derivative	MIC(μg/mL)	TI
DS-12341	1	1.536
DS-11630	2	0.188
5175128	2	0.025
DS-4609	4	0.5905
AS-63360	16	0.7625
5175126	>32	/
8015-2078	>32	/
7311-0001	>32	/
AN-970/40920790	>32	/

To minimize the toxicity of these derivatives, drug combinational assessment by checkerboard assay was performed. Partial synergistic antibacterial activities were observed between tiliquinol and certain conventional antibiotics (tetracycline, linezolid, ampicillin, doxycycline, and levofloxacin) (**Supplementary Figure 5**). The partial synergistic antibacterial activity was observed between the four derivatives (5175128, DS-4609, DS-11630, and DS-12341) and some traditional antibiotics (**Supplementary Figure 7**). Additionally, the synergistic antibacterial activity against *S. aureus* type strains ATCC 43300 was observed between 5175128 and TCY, as well as between DS-11630 and TCY. It was noteworthy that 5175128 and LZD had synergistic activity against different *S. aureus* type strains (**Figure 7C**). Collectively, derivatives of tiliquinol exhibited cytotoxicity, but it was suggested that the drug concentration could be an alternative to enhance its applicability.

## 4 Discussion

The increase rate of drug resistance among *S. aureus* is found to significantly outpace the *de novo* development of antibacterials. In recent years, host-directed therapies (HDTs) and multitarget drug design have emerged as alternative strategies to combat antibiotic resistance. HDTs modulate host immune pathways to reduce bacterial burden without directly selecting resistance (Zheng et al., 2026), but face challenges such as off-target effects, inter-individual variability, and potential toxicity. Multitarget drugs simultaneously inhibit several bacterial targets to delay resistance (Feng et al., 2023). However, the rational design of such compounds is technically demanding, requiring careful balancing of target affinities, physicochemical properties, and pharmacokinetic profiles, and most reported multitarget antibacterials remain in preclinical stages. In contrast, drug repurposing offers distinct practical advantages: repurposed agents already have established safety profiles, drastically shortening development timelines and costs. Moreover, many repurposed drugs, including tiliquinol, possess inherent multitargeting properties, providing resistance-delaying benefits without complex molecular design. Unlike HDTs, repurposed antibiotics also exert direct antibacterial activity, which is essential for rapidly clearing acute infections. Thus, while HDTs and multitarget design are valuable long-term strategies, drug repurposing represents a more pragmatic and immediately accessible approach to tackle multidrug-resistant *S. aureus*.

In this study, tiliquinol, an anti-amoebic drug, was demonstrated to exert effective antibacterial effects against MRSA and its biofilms both *in vivo* and *in vitro*, without inducing resistance. Mechanistic investigations revealed the dual antimicrobial targets by tiliquinol: PMF and peptidoglycan. In addition, tiliquinol is the one with the best antibacterial effect and the greatest antibacterial potential among its analogues.

It is important to note that no antibacterial activity of tiliquinol against Gram-negative bacteria has been observed. The difference may be attributed to structural variations in the cell walls of Gram-positive and Gram-negative bacteria. The cell wall of Gram-positive bacteria consists solely of a single layer of peptidoglycan, which provides a relatively weak barrier against foreign molecules. In contrast, the cell wall structure of Gram-negative bacteria is more complex, comprising the outer membrane, outer membrane proteins, and peptidoglycan among other components. lipopolysaccharides present within the outer membrane of Gram-negative bacteria have been shown to significantly contribute to drug resistance (Plumet et al., 2022). These structural differences likely elucidate the differential antibacterial activity exhibited by tiliquinol.

This study discovered that tiliquinol against MRSA by disrupting PMF and inhibiting peptidoglycan synthesis. Similarly, as reported by R. M. Birlutiu et al. (2025), oritavancin showed antimicrobial activity against MRSA through dual mechanisms involving depolarization of cell membranes and inhibition of peptidoglycan synthesis. Different from our study, oritavancin inhibited peptidoglycan synthesis by binding to D-alaninyl-D-alanine terminals (Wang et al., 2021). However, tiliquinol prevented UDP-N-acetylmuramyl (UDP-MurNAc) synthesis by influencing on key enzymes. Antibacterial agents with diverse targets may mitigate drug resistance risks (Birlutiu and Birlutiu, 2025), such as tafenoquine, but the MIC of tafenoquine is higher than that of tiliquinol against MRSA (She et al., 2024). PMF refers to the electrochemical gradients across bacterial cell membranes, which is critical for biofilm formation and energy metabolism (Yang et al., 2023). The disruption of the proton gradient by tiliquinol led to a breakdown of the PMF resulting in cell damage or even cell death. Meanwhile, tiliquinol also affected peptidoglycan synthesis by the inhibition of key enzymes. Molecular docking indicated that tiliquinol formed strong interactions with the active site of key enzymes by hydrogen bonds and hydrophobic interactions. Molecular components of the MRSA bacterial cell wall, including amyloid proteins, peptidoglycans, and lipoteichoic acids, are also crucial for antimicrobial tolerance as well as biofilm formation, maturation and dispersion, making them potential targets against biofilm-related infections (Ahn et al., 2018; Katsipis and Pantazaki, 2023). Therefore, tiliquinol presents novel mechanisms for the treatment of MRSA and its biofilm-related infections.

*S. aureus* is one of the most common pathogenic bacteria in PJIs. MRSA has become the primary concern in the treatment of PJIs due to antibiotic resistance (Kraus et al., 2024). PJIs are one of the most serious complications after total joint arthroplasty, leading to multiple revision surgeries, prolonged antibiotic therapy, and high patient morbidity and also increased healthcare costs (Sigmund et al., 2025). Therefore, the pursuit of new treatment options has been deemed critically urgent. Current standard therapies, including VAN, linezolid, and daptomycin, are often limited by factors such as inadequate bone permeability, toxic side effects, the emergence of drug resistance, and restricted efficacy against biofilms (Zhao et al., 2023). In this study, tiliquinol demonstrated significant efficacy in treating PJIs caused by MRSA, which could probably due to its notable anti-biofilm effect.

As reported by Soffer et al. (1983), the patient administered overdose of tiliquinol-tilbroquinol (80 mg and 200 mg daily, respectively, continuously for four years) may cause neurotoxicity. Notably, the treatment was about 50 times longer than the period recommended by the manufacturers, suggesting that neurotoxicity resulted from cumulative compound exposure combined with significant overdose. As an antimicrobial agent against MRSA, tiliquinol could be well-tolerant. Because antibiotics are generally prescribed for relatively short durations. In addition, due to the high structural similarity between tilbroquinol and tiliquinol, the cumulative toxic effects of their combined use in the body may be amplified.

Tiliquinol had effective antimicrobial activity in the skin abscess model, despite unsatisfactory blood concentration. The transport of highly plasma protein bound drugs into deeper dermal tissues occurs several orders of magnitude faster than predicted by passive dermal diffusion. When applied topically, highly plasma protein−bound drugs rely on convective transport to directly reach deep tissues (Dancik et al., 2012). A similar phenomenon has been observed with subcutaneously administered bunamidine hydrochloride in the treatment of skin and soft tissue infections: although plasma concentrations are suboptimal, the drug demonstrates favorable local antibacterial activity (She et al., 2025). Although tiliquinol demonstrates potent antimicrobial activity and robust efficacy in local infection models, its high plasma protein binding and the neurotoxicity risk to possible drawbacks for systemic use. However, after structural optimization, tiliquinol is expected to become a feasible antibacterial agent.

## Data Availability

The data presented in the study are deposited in the Mendeley Data repository https://data.mendeley.com/, accession number DOI: 10.17632/mwyjxd4cdm.2.
