# Supplementary material for: Drug repurposing: a dual-mechanism antibiotic combats MRSA and its high resistant phenotypes
Source: Front Cell Infect Microbiol. 2026 May 28;16:1851763. doi: 10.3389/fcimb.2026.1851763 (PMC13254916; doi:10.3389/fcimb.2026.1851763)

**Supplementary Information**

**1 Supplementary Table**

**Supplementary Table S1** Primers of cell wall-related genes used for qRT-PCR

| Genes | Forward (3’→5’) | Reverse (3’→5’) | Product size (bp) |
| --- | --- | --- | --- |
| *uppP* | CGTCGGCTTATTCCAATGTCT | CGCCATAATAAACGTAAAGTCGG | 124 |
| *cobQ* | AGCCTGATGAAAGAATGAATCCC | CACTCAACCTTCACCCCGTA | 127 |
| *mrcA* | ATTGCTTTCTCTGCGCCGTA | CGATAACCGTCTTCATAGGCAC | 89 |
| *femB* | ATGGCGAAAGAAAACAAATCCG | CGTCCGTTTTGCTCAATTCACT | 115 |
| *murE* | GAACACTACGAAGCCGCGAAA | CCACTTCATCGTCGGCGTT | 82 |
| *murF* | AAATTGGAATGCCGTTGACT | TCGCCAATGTTTGTGATGACC | 145 |

**2 Supplementary Figures**

**Supplementary Figure Legends**

**Figure. S1.** Post-antibiotic effect (PAE) of tiliquinol against *S. aureus*, with VAN serving as a control.

**Figure. S2.** The anti-biofilm effect of tiliquinol against *S. epidermidis.* The inhibitory (left) and eradication (right) effects of tiliquinol against the *S. epidermidis* RP62A biofilm were determined by crystal violet (CV) and XTT reduction assay, respectively.

**Figure. S3.** The membrane permeability determination of *S. aureus* by tiliquinol. Tiliquinol did not increase the membrane permeability of *S. aureus* as determined by SYTOX Green.

**Figure. S4.** Docking patterns of tiliquinol with five different cell wall enzymes.

**Figure. S5.** Tiliquinol had partial synergistic activity with traditional antibiotics. Tiliquinol had partial synergistic activity with daptomycin, tetracycline, ampicillin, linezolid, ciprofloxacin, amikacin, doxycycline and levofloxacin.

**Figure. S6.** *In vivo* toxicity by tiliquinol. (A) Quantification of liver biomarker glutamic-pyruvic transaminase (ALT). (B) Quantification of renal biomarker reaction products with formaldehyde (Urea). (C) Platelet (PLT) quantification. (D) White blood cell (WBC)-related parameters of WBC counting (left panel) and neutrophils proportion (N%, right panel). (E) RBC-related parameters of RBC counting (left panel) and hemoglobin (HGB) quantification (right panel). (F) H&E staining of organs.

**Figure. S7.** The chessboard method was used to determine the interactions between the selected four analogues (5175128, DS-4609, DS-11630, and DS-12341) and antibiotics (AMP, Gentamicin, and LZD).

**Supplementary Figure 1**


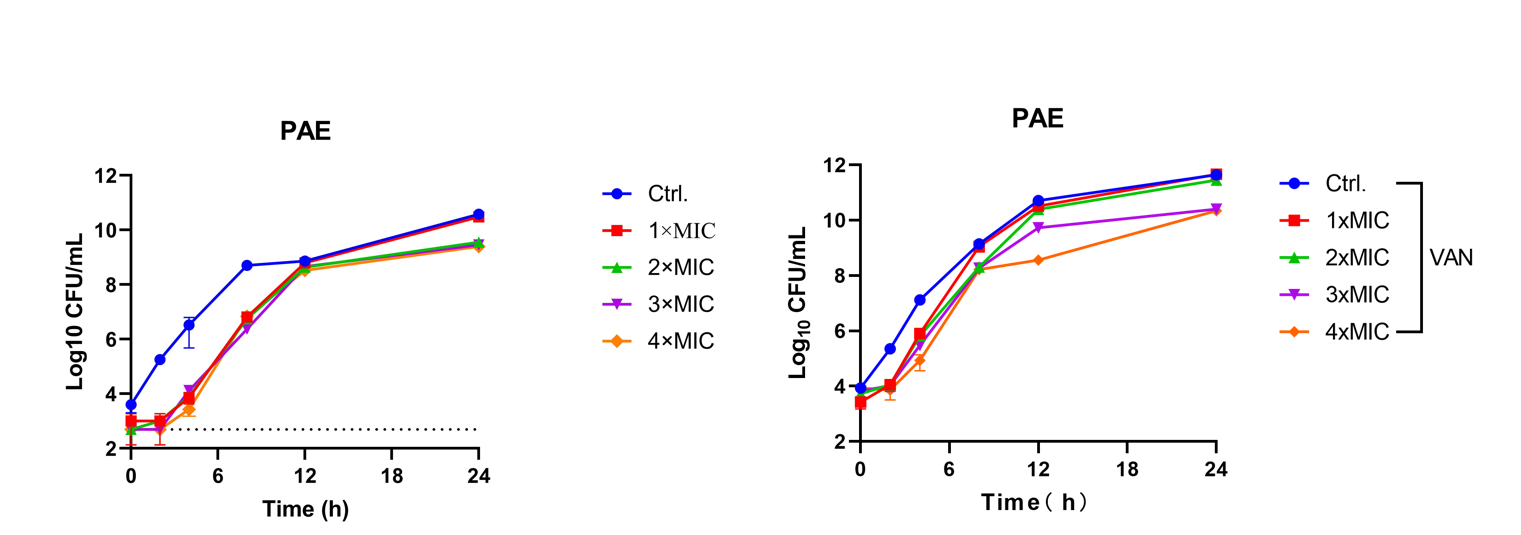


**Supplementary Figure 2**


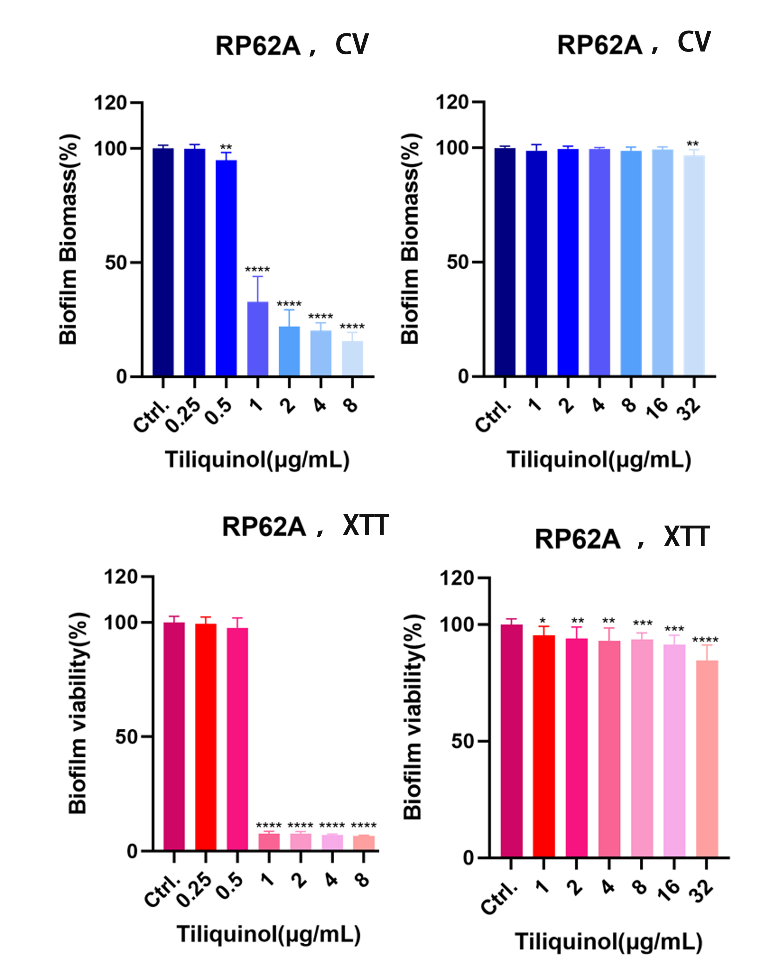


**Supplementary Figure 3**


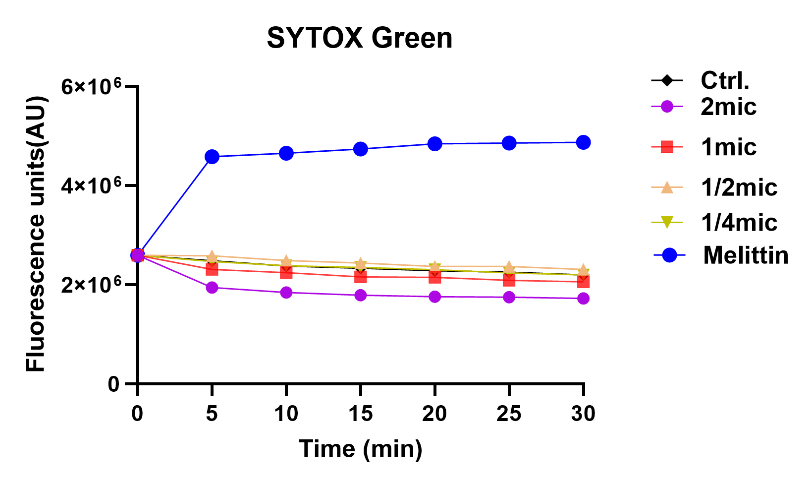


**Supplementary Figure 4
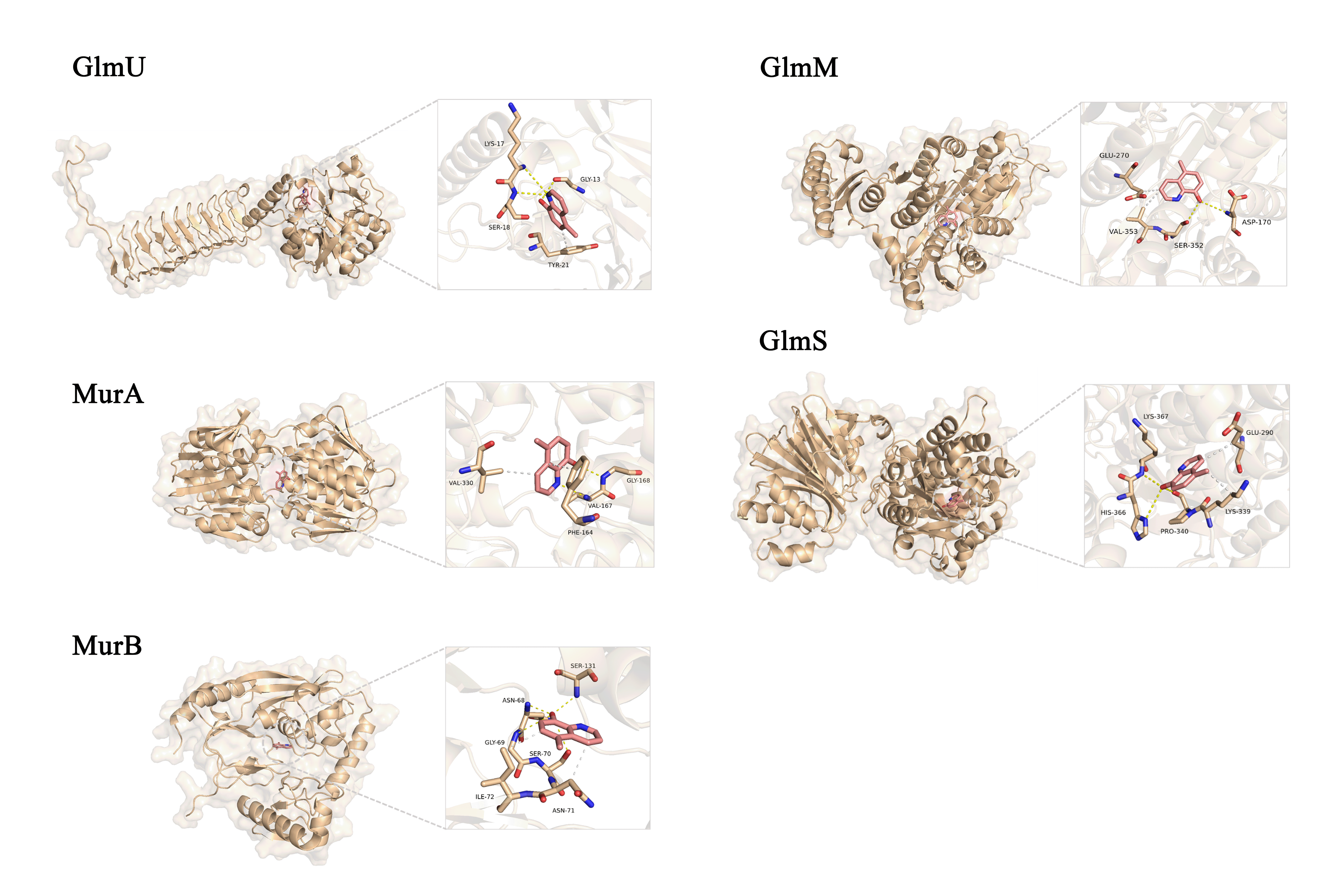
**

**Supplementary Figure 5**


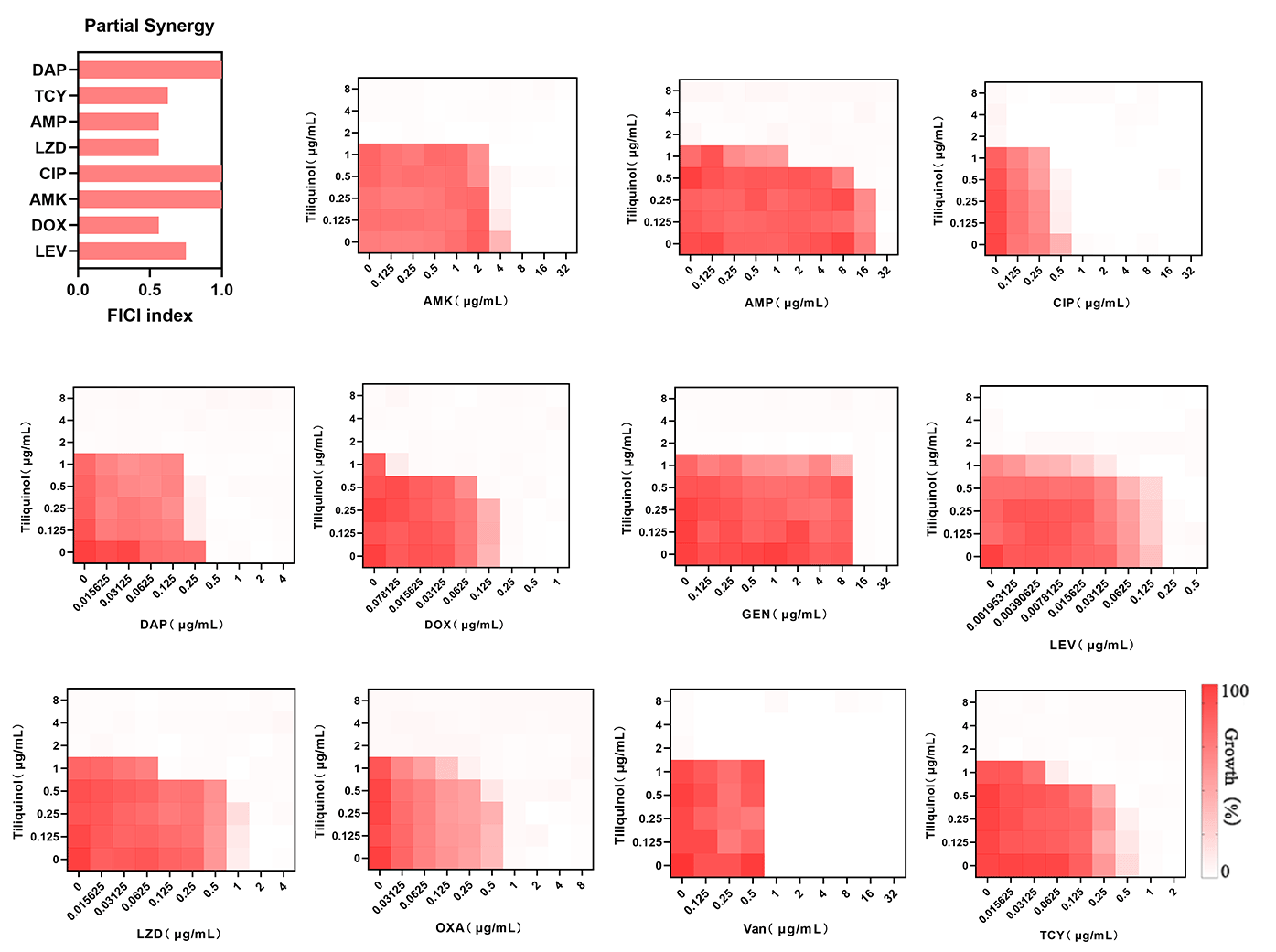


**Supplementary Figure 6**


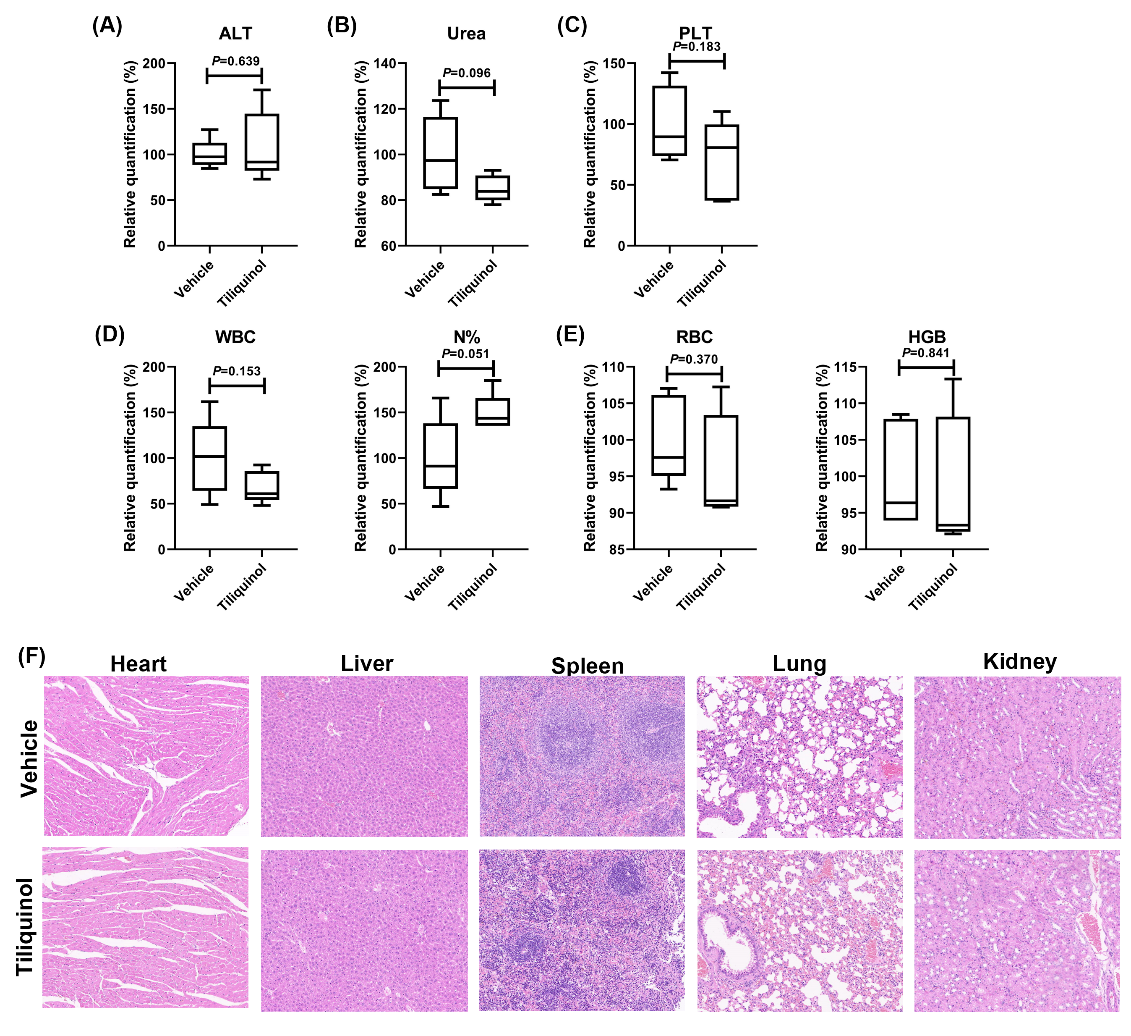


**Supplementary Figure 7**


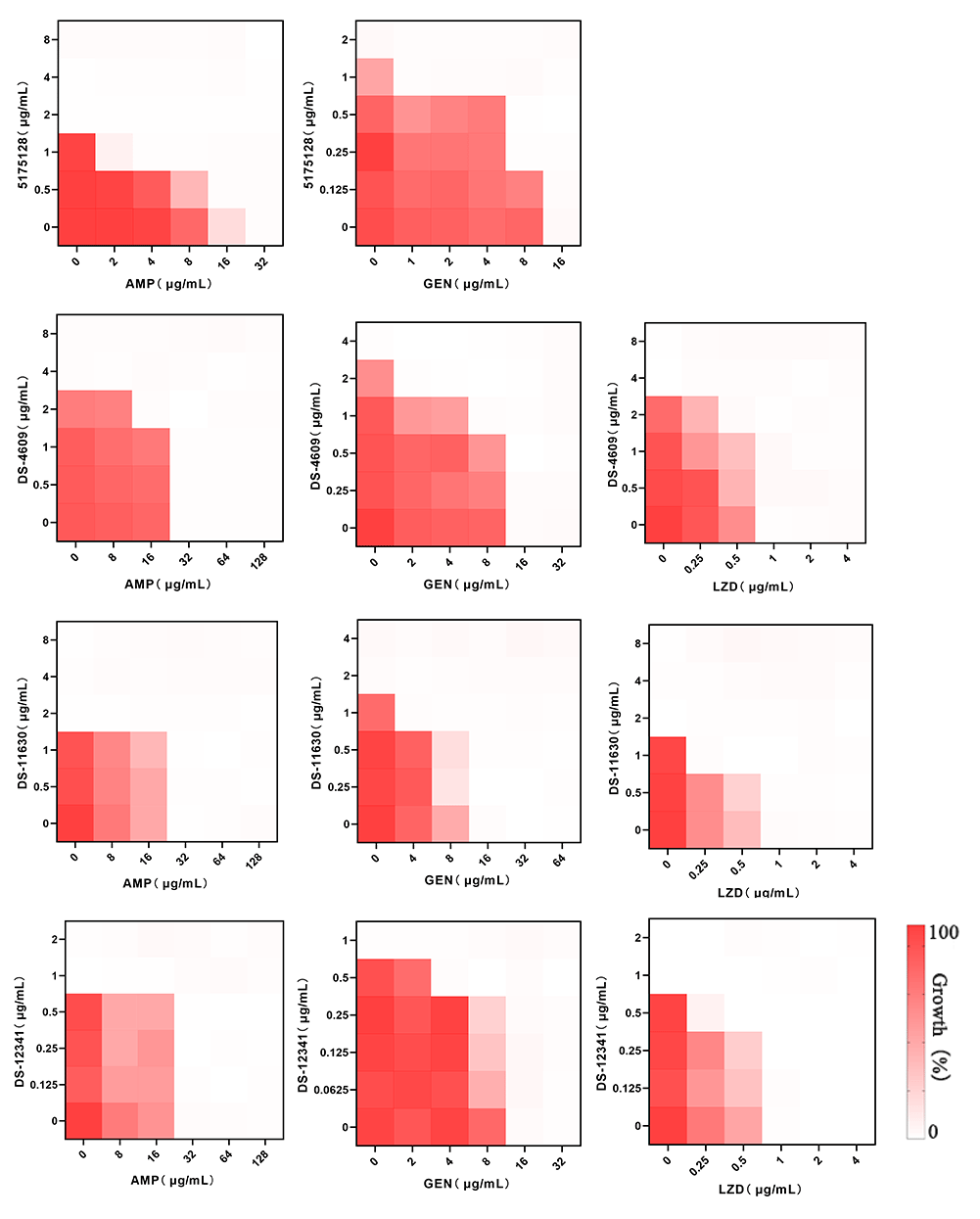

Supplement: Supplementary file 1 [file Table1.docx]
